# Engineering Biological Tissues from the Bottom-Up: Recent Advances and Future Prospects

**DOI:** 10.3390/mi13010075

**Published:** 2021-12-31

**Authors:** Xiaowen Wang, Zhen Wang, Wenya Zhai, Fengyun Wang, Zhixing Ge, Haibo Yu, Wenguang Yang

**Affiliations:** 1 School of Electromechanical and Automotive Engineering, Yantai University, Yantai 264005, China; WXW2435461356@163.com (X.W.); wangzhenqh@163.com (Z.W.); fywang0213@sina.com (F.W.); 2 School of Energy and Materials Engineering, Shandong Polytechnic College, Jining 272000, China; zhaiwenya20@163.com; 3 State Key Laboratory of Robotics, Shenyang Institute of Automation, Chinese Academy of Sciences, Shenyang 110016, China; gezhixing@sia.cn (Z.G.); yuhaibo@sia.cn (H.Y.)

**Keywords:** bottom-up tissue engineering, drug screening, modular assembly, micro-fabrication

## Abstract

Tissue engineering provides a powerful solution for current organ shortages, and researchers have cultured blood vessels, heart tissues, and bone tissues in vitro. However, traditional top-down tissue engineering has suffered two challenges: vascularization and reconfigurability of functional units. With the continuous development of micro-nano technology and biomaterial technology, bottom-up tissue engineering as a promising approach for organ and tissue modular reconstruction has gradually developed. In this article, relevant advances in living blocks fabrication and assembly techniques for creation of higher-order bioarchitectures are described. After a critical overview of this technology, a discussion of practical challenges is provided, and future development prospects are proposed.

## 1. Introduction

Tissue engineering is an emerging technology, which combines cell biology and materials science to construct tissues or organs in vitro [1,2,3,4,5,6,7]. The history of tissue engineering can be traced back to the 1980s, when Professors Joseph P. Vacanti and Robert Langer first proposed the research and exploration of tissue engineering [5]. In the early stages, scientists successfully used tissue engineering technology to create human auricle cartilage with the skin of mice [4], which symbolizes that tissue engineering technology can form tissues and organs with complex three-dimensional spatial structures for clinical application [2,8,9,10,11,12,13,14]. Current tissue engineering techniques can be used to reconstruct a variety of tissues, such as muscle, bone, cartilage, tendon, ligament, blood vessels and skin [13,15]. These tissue engineering developments will change traditional disease treatment and drug screening. Methods of tissue engineering have been divided into top-down and bottom-up.

In top-down tissue engineering, cells are implanted into 3D scaffolds to simulate the physicochemical and biomechanical signals of the extracellular matrix(ECM). The 3D scaffolds used usually replicate the overall size and shape of the target tissue rather than the complex internal structure. In this method, cells attach, proliferate, and eventually fully attach to the 3D biodegradable scaffold, eventually forming ECM. Currently, researchers have designed scaffolds that enable biomaterials to better reproduce the internal microscopic environment, mechanical biology and biomolecular signaling. However, this method still has big challenges in clinical application. Firstly, there are many difficulties in the process of cell fixation on the scaffold, resulting in low density of cell seeding and uneven distribution on the scaffold. Secondly, in the bodies of persons, cells are closed to blood vessels, which supply nutrients and oxygen to tissues and remove waste products and carbon dioxide. The lack of basic nerves and blood vessels and the lack of oxygen and nutrients in the inner area are the main challenges in the use of scaffolds for tissue culture. To overcome this shortcoming, it is particularly important to design a vascular network with similar functions. One approach is to implant endothelial cells and smooth muscle cells into scaffolds and induce them to release growth factors that promote angiogenesis in scaffolds. The main challenge with this approach is that during the process of producing blood vessels, many cells may lose their ability to survive due to a lack of oxygen and nutrients. Another approach is to prevascularize the tissue before implantation, by creating artificial microvessels inside the scaffold that bind to the host’s blood vessels when implanted. The main challenge with this approach is the difficulty of creating such a controlled structure and ensuring its proper function after implantation. To sum up, although progress in biocompatibility stent manufacturing technology has been made, based on the support of the top-down method, it is usually only used in cultured cells of the anatomy of the relatively few species, and simple organization, such as skin and cartilage tissue, cannot simulate human tissue (such as renal unit, lobular, islet) repeating units in the modular design [16].

**Figure 1:**
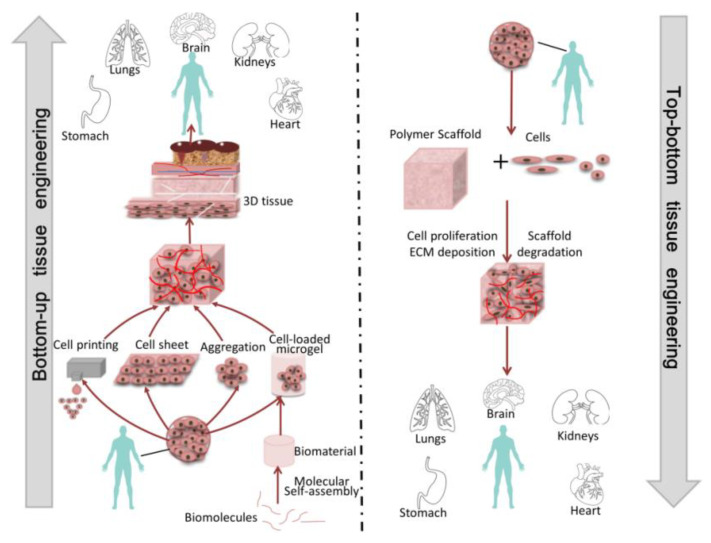
The schematic diagram of bottom-up tissue engineering and top-down tissue engineering. In the bottom-up tissue engineering approach, there are multiple ways to create modular units and then assemble those units into specific engineering organizations. In the top-down tissue engineering approach, cells are seeded on the biomaterial scaffold, which is then cultured until the scaffold is full of cells to create a new engineered tissue.

Over the years, bottom-up tissue engineering was developed to take the place of top-down tissue engineering [17,18]. This method is usually used to fabricate function units, such as cell aggregations and cell sheets, which constructs bigger tissues or organs by using the modular assembly approach [1,11,16,19,20]. Additionally, this technology aims to create modular tissues with physiological microstructure characteristics, and then provide more guidance at the cellular level to guide tissue morphogenesis [19]. Unlike top-down tissue engineering, bottom-up tissue engineering has unique design flexibility and allows module units to be combined through different methods, including self- or guided/programmed cell assembly, through which the spatial distribution of module units can be controlled to construct tissues or organs (Figure 1). The advantages of bottom-up tissue engineering are obvious. Firstly, we only need to isolate the dominant cells in the target tissue, enlarge them, and then use them for tissue reconstruction without the need for other cells, such as nerve cells and blood vessel cells. Secondly, the use of this technology can avoid the degradation of cell activity due to the use of scaffolds. In view of the above advantages, bottom-up tissue engineering has been widely used in clinical medicine.

In this paper, various module units for bottom-up tissue engineering are reviewed, and their manufacturing and assembly methods are introduced. The present applications of bottom-up tissue engineering are also introduced. After a critical overview of this technology, a discussion of practical challenges is provided, and future development prospects are proposed.

## 2. Module Manufacturing

In living organisms, many tissues or organs are composed of repetitive functional modules, such as liver lobules, muscle fibers, and so on. Hepatic lobules are the basic functional unit of liver, which are usually hexagonal prismatic, and there are about 500,000 to 1,000,000 hepatic lobules in an adult. The muscle cell is the basic component unit of skeletal muscle, which are arranged in bundles to form skeletal muscle.

The top priority of bottom-up tissue engineering is to construct functional module units, which are usually composed of microscale cell-carrying hydrogels (such as polyethylene glycol, collagen, polyethylene glycol diacrylate, and gelatin-methacrylic anhydride [9,21,22,23]). At present, there are six methods commonly used to prepare cell microgel modules, including emulsification, microchannels, microfluidic technology, bioprinting technology, and the liquid bridge manufacturing module units method [24] (Table 1).

### 2.1. Emulsification Method

**Figure 2:**
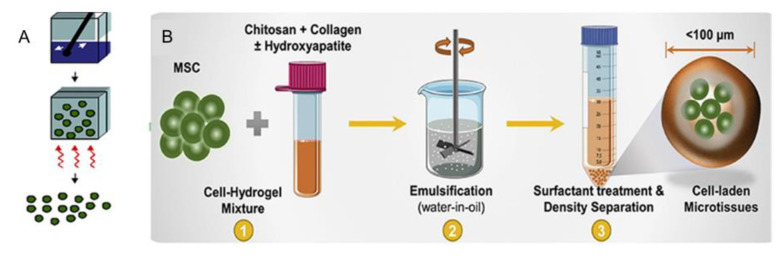
(**A**) Cell-loaded microgel module units are fabricated by the emulsification method (reproduced with permission from the Reference [25]). (**B**) Preparation of chitosan-collagen microgels supported by mesenchymal stromal cells (MSC). The first step: Mesenchymal stem cells (MSC) are added into a mixed solution of chitosan, collagen and hydroxyapatite to form a hydrogel mixed solution that contains mesenchymal stromal cells, which is called a heterogeneous mixture. The second step: The multiphase mixture is stirred to produce small droplets of hydrogel precursors within the organic phase. The last step: The droplets are cross-linked by various mechanisms to form spherical microgels. Finally, cell-loaded microgel modules can be obtained. (Reproduced with permission from the Reference [23]).

The emulsification method has been widely used to prepare cell-loaded microgel modules [25]. In this method, an emulsion will form with the mixture of two incompatible liquids (Figure 2A). The first step of this method is to stir the multiphase mixture to produce small droplets of hydrogel precursors (Figure 2B), whose size is controlled by the degree of mechanical agitation, viscosity of each phase, and the presence of surfactant [23]. Additionally, the spherical microgels are then formed by various cross-linking mechanisms, which can be used for immunoisolation, carriers in bioreactors, or analysis of stem cell biology applications by adding cells in the water phase. However, emulsification technology tends to be limited to spherical microgels, which typically have a wide distribution of sizes, so it is hard to control the final shape of microgels.

### 2.2. Optical-Based Fabricating Method

**Figure 3:**
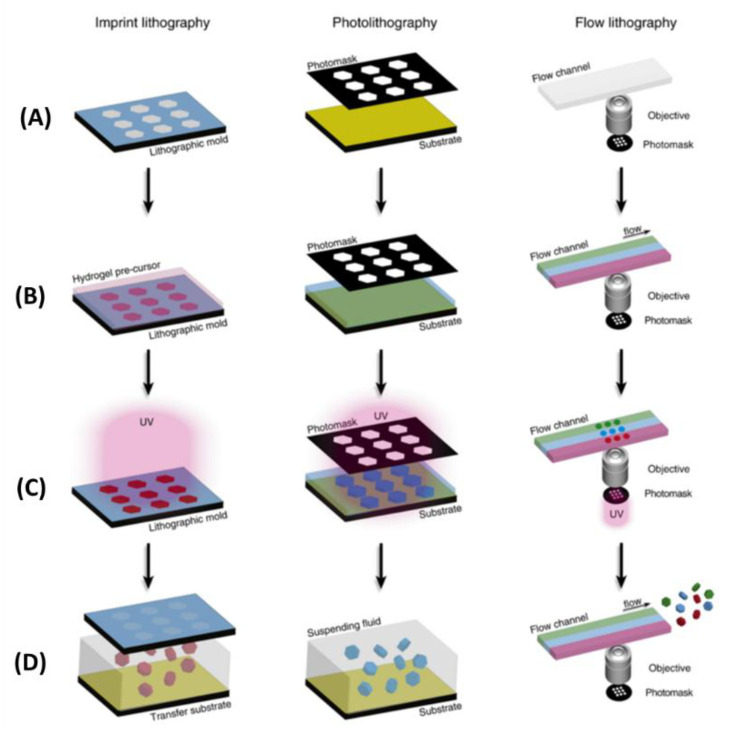
Schematic diagram of hydrogel colloid synthesis by photographic method. Embossed lithography (left), lithography (middle) and flow lithography (right) are shown. The steps of optical graphics are as follows: (**A**) Make a template. (**B**) The fluid reservoir is filled with hydrogel precursor fluid. (**C**) Hydrogel colloid is synthesized by a synchronous mode transfer and crosslinking reaction. (**D**) Recovery of units from fluid reservoirs. (Reproduced with permission from the Reference [27]).

The optical-based fabricating method is another way to obtain microgel modules [27], and the steps of this method are shown as follows: Firstly, cell suspension is mixed with photopolymerizable hydrogel. Then, ultraviolet light is projected onto a specific position in the mixture through a photomask, and the photopolymerizable hydrogel exposed to UV light begins to cure. Finally, the hydrogel prepolymer without crosslinking is washed, and the hydrogel modules with a designed pattern are formed [26,27]. Through this method, we can obtain many modular units with customized shapes and sizes. Imprint lithography (left), lithography (middle) and flow lithography (right) are three common system based on optics fabricating microgel modules (Figure 3). In the photolithography and flow photolithography method, the mask is a light pattern (or other radiation) which induced the material to be synthesized. In most cases, the microgels obtained by this method were two-dimensional and the thickness of the microgel was determined by the height of the fluid. Du et al. used the optical-based fabricating method to construct blood vessels by bottom-up tissue engineering. In this process, they first used this technique to fabricate microgel modules with internal microchannels, and assembled these modules into three-dimensional tubular structures in a dual-phase reactor by the physical forces and fluid shear generated by sliding needles. Finally, endothelial cells and smooth muscle cells were integrated into the assembled structure to construct a three-dimensional vascular system [31]. In addition, with the development of projection technology, digital micromirror devices (DMDs) were used to produce a light pattern instead of traditional masks [45]. Using a DMD as the mask, the pattern can be designed and changed with flexibility and ease. The modules created by this method have highly complex and interrelated shapes compared with imprint lithography. Yang et al. proposed a system that combined high-throughput manufacturing with flexible assembly of tiny tissues. They used digital micromirror devices (DMDs) to rapidly fabricate cell-loaded hydrogel modules, and finally flexibly assembled these modules by using the power of dielectric electrophoresis. This research shows the optical-based fabricating method is very fast. Although the optical-based fabricating method is programmable, fast, and easy to operate, the crosslinking materials and ultraviolet radiation may affect cell function, and this method is only suitable for photosensitive materials.

### 2.3. Micromolding Method

**Figure 4:**
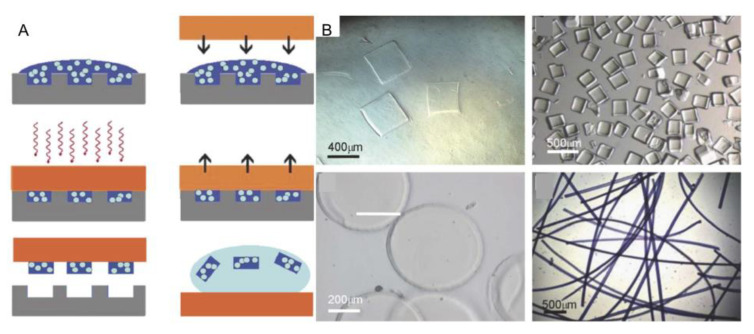
(**A**) The steps of the micromolding method: First of all, the cells are suspended in a hydrogel precursor solution, and then the mixed solution is molded by using a PDMS stamp. Then the hydrogel precursor solution is photocrosslinked to form microgel modules. Finally, the mold is removed to create a set of micromolded microgel module units that can be collected into the solution by simple cleaning. (**B**) Microgel modules can be molded into various shapes (such as square prisms, disks and chords) with different kinds of prepolymer solutions. (Reproduced with permission from the Reference [39]).

Recently, researchers have developed a technique to uniformly produce cell-loaded hydrogels whose size and shape can be controlled, which is known as the micromolding method [39]. Firstly, the cells are suspended in a hydrogel precursor solution, and then the mixed solution is molded by using a PDMS stamp. Then the hydrogel precursor solution is induced to be crosslinked to form microgel modules. Finally, the mold is removed to create a set of micromolded microgel modules which can be collected into the solution by simple cleaning (Figure 4A) [39]. At first, researchers usually use this method to produce spherical, clubbed, and circular modules. However, now, an increasing number of microgel modules with complex shapes can be obtained by this method (Figure 4B). Charnley et al. performed dynamic analysis of individual adult stem cells in microporous plates, analyzing their shape, stiffness, and dimension. They attempted to control and manipulate the growth of stem cell colonies in microporous plates and the formation of embryoids in microporous plates. Through these experiments above, they proved that the micromolding method has great advantages, and it has been widely used in cell biology [40]. The flexibility of this method seems poor due to limitations by the mold’s shape, and these module units may be destroyed in the demolding process.

### 2.4. Microchannel Method

**Figure 5:**
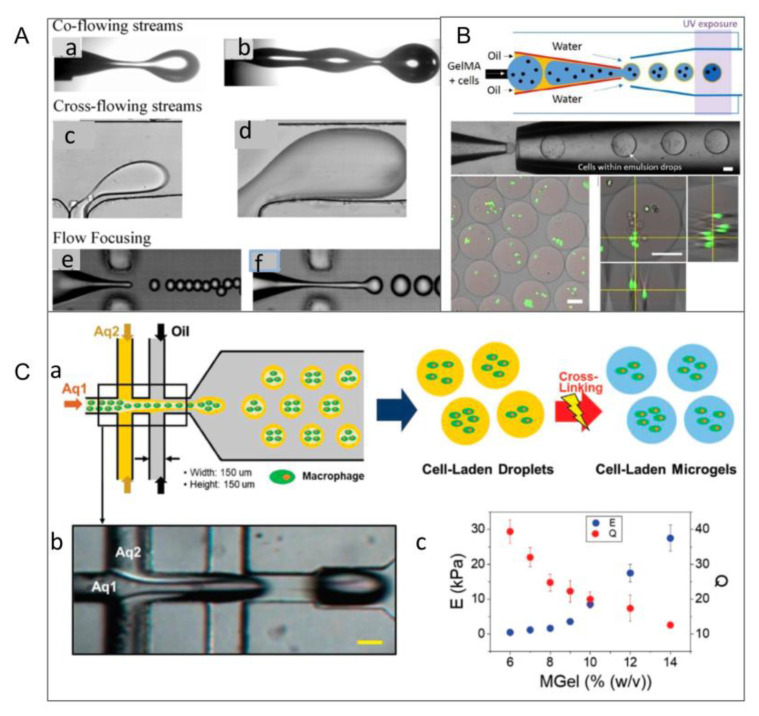
Illustration of the three main microfluidic geometries used to format droplet formations. (**A**) (**a**) Co-flow, (**b**) cross flow at t-junction, (**c**) elongating flow in focusing geometry. (Reproduced with permission from the Reference [33]). (**B**) Schematic diagram of producing cell-filled microgels. Live mammalian cells are embedded in thin-shell double-emulsion droplets and form cell-loaded microgels from photocrosslinked polymers (up). The optical images of cell encapsulation (bottom). (Reproduced with permission from the Reference [33]). (**C**) (**c**) Schematic diagram of prepared macrophage gel microgel controlled by double-flow focused microflow. (**b**) Core (Aq1) and shell (Aq2) that are delineated flow during droplet formation. (**c**) Elastic modulus (E) and swelling ratio (Q) of photocrosslinking of microgels with different concentrations. (Reproduced with permission from the Reference [34]).

The microchannel method produces module units by extruding and molding through microtubules or microchannels. There are three main types of the microchannel method, known as liquid emulsification, piezoelectric injection, and mechanical injection. In liquid emulsification, the consistency of the cell-loaded microgel size is mainly determined by the microfluidic emulsification process (Figure 5A). There are typical droplet breakup events in the three main microfluidic geometries. Breakup in co-flowing streams is shown in (a) dripping and (b) jetting modes. Breakup in cross-flowing streams is shown in (c) unconfined and (d) confined T-junction geometries. Flow-focusing geometries lead to breakup in elongational flows in (e) dripping and (f) jetting modes. Two kinds of incompatible liquids can form monodisperse emulsion droplets through total flow to control the size of droplets by changing the flow rate of the liquid (Figure 5B). With further development of this technology, Huang et al. developed a double-flow focused microflow device to fabricate modular units (Figure 5C) [34]. The droplets usually aggregate into a sphere to minimize the free energy of the surface, and by considering hydrodynamics and thermodynamics, some multiphase droplets with complex structures can be easily obtained by microchannel technology. The figure below shows the delineated flow of the core (Aq1) and shell (Aq2) during droplet generation (Figure 5C(b)). Finally, cell-loaded microgels were generated by photocrosslinking (Figure 5C(a)) [32]. In addition, modules with different size distribution and composition can also be efficiently fabricated based on piezoelectric injection and mechanical injection. Armada-Moreira et al. used this technique to produce different types of non-spherical particles, including wrinkled particles, multinucleated particles, and other particles with controllable shape and size. This research also showed that non-spherical particles were ideal models for exploring details of different shapes in cell simulation and biomedicine [46]. Yamadadengren et al. also used the technology to produce hydrogel fibers with highly complex cross-sectional morphology, strip hydrogel sheets, and yarn spherical hydrogel beads [47].

In contrast to other methods of building module units, the microchannel method can realize precise adjustment of spatial position of modular units, which provides convenient conditions for real-time assembly of modular units. However, precisely designed microchannels and complex devices are essential in this technology.

### 2.5. Bioprinting Technology

**Figure 6:**
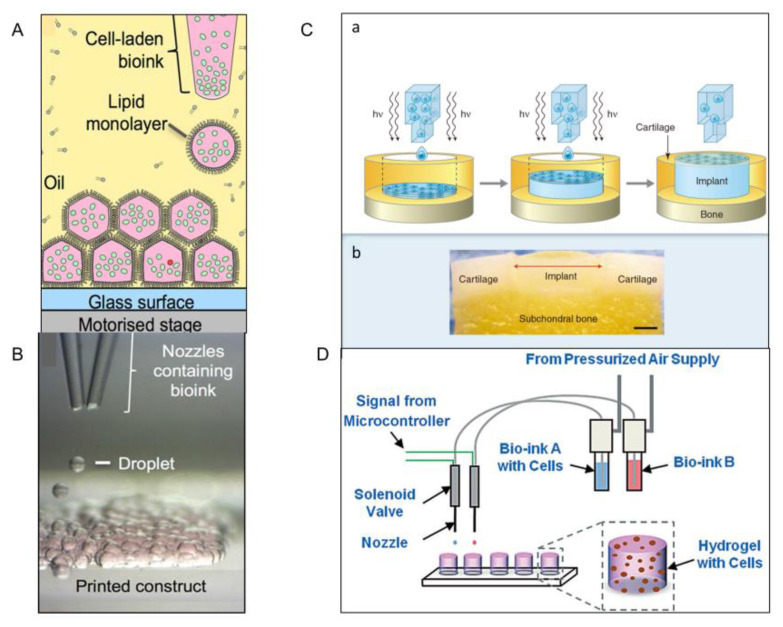
(**A**) Printed schematic diagram of a modular unit containing cells. The nozzles spray bioink droplets containing cells into oil containing grease. The droplets are positioned by the programmed motion of the oil container. The droplet condenses by forming a lipid bilayer at the droplet interface. (**B**) The glass nozzles contain two kinds of cells, and the bioink containing two types of cells is ejected from the two glass nozzles. (Reproduced with permission from the Reference [42]). (**C**) (**a**) Schematic diagram of the similar structure of bioprinted cartilage by combining inkjet printing with a solution of polyethylene glycol dimethacrylate (PEGDMA) containing suspended cells in a simultaneous photopolymerization process. (**b**) After a few days of culture, the printed structure seemed to have integrated into the surrounding tissue, which demonstrated the feasibility of the method. (Reproduced with permission from the Reference [41]). (**D**) Honeycombed printing process based on inkjet technology. (Reproduced with permission from the Reference [42]).

Bioprinting is an emerging technique for making living tissue that allows cells to be arranged in a predetermined three-dimensional structure [41,43]. Here, a low-cost process is proposed that uses 3D-printed droplets containing mammalian cells to produce firm, patterned structures in oil that can be transferred to culture medium (Figure 6A) [44]. At present, this structure is limited to two cell types, but in the future, the printer may be adapted to include a multi-nozzle dispenser, thereby increasing the number of moldable cell types (Figure 6B). In addition, combined with an extrusion-based system, clinical-grade tissues with complex 3D cellular characteristics can be rapidly manufactured, which cannot be achieved by extrusion or droplet-printing alone. 

Derby et al. used an aqueous solution of polyethylene glycol dimethacrylate containing suspended chondrocytes and printed it into an osteochondral defect model. After a few days of culture, the printed structure integrated into the surrounding tissue. Although this study shows great promise for the technique, the potential cytotoxicity of photoinitiators must be addressed in the general use of photoinitiated cross-linked cell suspensions (Figure 6C) [42]. In addition, Lic et al. developed hydrogels offering the possibility of constructing three-dimensional structures of cells (Figure 6D). The bioink can also enable them to maintain stable and uniform cell suspension, preventing cell sedimentation and aggregation. Using bioprinting to obtain cell-loaded microgel module units is very fast and precise, in which the survival rate of cells can be highly protected. However, the high cost of equipment and the limited variety of solutions cannot be ignored.

### 2.6. Liquid Bridge Method

**Figure 7:**
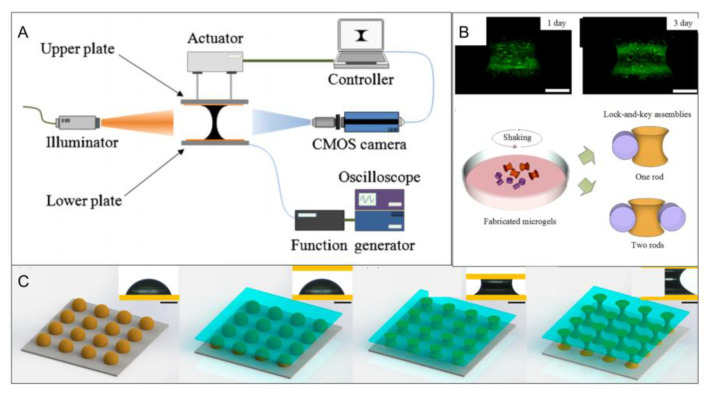
(**A**) Schematic diagram of the liquid bridge. The movement of the upper plate is controlled by a pneumatic actuator, and the lower plate is fixed during the experiment so that the droplets remain stable until they come into contact with the upper plate. Then the light source creates a brightness difference between the liquid and gas regions to make the droplet boundary clearer. The initial droplet and the droplet-splitting process are captured by a complementary metal oxide semiconductor camera. (Reproduce with permission d from the Reference [36]). (**B**) Fluorescent images of live/dead staining of cells in hydrogel at t = 24 h and t = 72 h (green represents live cells and red represents dead cells) (top left corner); statistical results of cell viability (top-right corner); schematic diagram of directional assembly of dumbbell microgels and rod microgels (bottom-left corner); phase contrast image of two microgel components (bottom-right corner). (Reproduced with permission from the Reference [38]). (**C**) Flow chart of preparing a microgel module by using a liquid bridge phenomenon. At first, the base material is stuck onto the upper part of the lifting platform with double-sided tape. Next, the joint is loosened and the arm is moved until the upper plate is just parallel to the lower plate, which ensures that the two plates are parallel between the liquid bridge. The joint is fixed, another substrate is placed below, and a drop of gel precursor is placed on the substrate. Then, a liquid bridge is formed between the two bases by moving the arm. The knob is then adjusted so that the liquid bridge is stretched or compressed at a low speed to achieve the desired shape. (Reproduced with permission from the Reference [36]).

In addition to the above five methods, researchers stretched the hydrogel precursor droplets to form a liquid bridge between the two substrates by adjusting the distance between the two substrates and their hydrophobicity, where microgels with custom microstructures such as barrels, dumbbells or funnels can be obtained (Figure 7) [38]. Additionally, they demonstrated the applicability of the method to temperature-, light- and chemical-sensitive hydrogels [37,38]. In later studies, researchers controlled the volume of the hydrogel precursor by using microliter syringes, and different sizes of microgels were obtained. However, evaporation becomes the main problem with decreases of liquid volume that lead to deformation of the liquid bridge.

## 3. Module Assembly

In bottom-up tissue engineering, the assembly of units is the key to the formation of a functional organizational structure [8,12,45,48,49,50,51]. At present, the assembly of module units is faced with the following problems: (1) Preparing materials with biomimetic mechanical properties; (2) building a complex organization with a controllable microstructure; (3) forming a network of functional blood vessels; (4) compatibility. The assembly methods are shown in Table 2.

### 3.1. Acoustic Assembly

**Figure 8:**
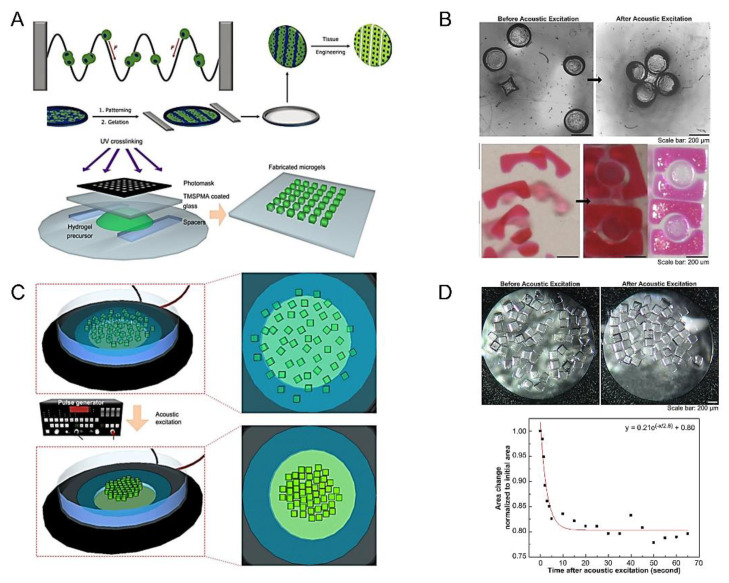
(**A**) Acoustic radiation force (F) can shift the cells toward the pressure node of an ultrasonic standing wave. (Reproduced with permission from the Reference [79]). (**B**) Image of key microgels before and after acoustic excitation. The circular microgels (diameter 200 mm) are connected with the star microgel, and the 200 mm circular microgel is wrapped in the 1 mm square microgels. (Reproduced with permission from the Reference [73]). (**C**) The steps of acoustic assisted assembly: Firstly, microgels are prepared by photolithography. Then, an acoustic assembler is used to assemble microgels in the droplet. Under the acoustic excitation, the microgels are assembled by using transducers. (**D**) State of microgel before and after acoustic-assisted assembly. The relation of normalized area with acoustic excitation time (bottom). (Reproduced with permission from the Reference [73]).

Sound waves can capture, move, sort and manipulate cells and particles in a fluid environment. In the beginning, acoustic excitation assembly was raised by Xu et al. which is used to assemble module units for bottom-up tissue engineering [73,74,75,76,77,78]. In this technology, acoustic radiation force (F) could shift cells towards the pressure node of ultrasonic standing waves, which indicated that an acoustic wave can manipulate cell positions. Researchers then used ultrasonic standing waves to guide the assembly of myoblasts in collagen-based hydrogels (Figure 8A) [79]. In the development of acoustic assembly, the assembly chamber (such as a petri dish) is placed on top of a piezoelectric transducer, which is stimulated by a pulse generator to deform and vibrate, producing sound waves. As a result, module units scattering throughout the assembly room begin to move and gather to form a dense gel mass (Figure 8C) [73]. This technique can not only realize single-layer assembly of modules with various shapes (such as square, Z, zigzag, lock and key) (Figure 8B) [73], but also construct 3D multi-layer structures by the layer assembly method (Figure 8D). The distributed microgels are first assembled into monolayers by using acoustic excitation, and then the monolayer assembly is stabilized by photocrosslinking. The new microgel is introduced into the monolayer surface of the assembly to form the second layer of the assembly. Finally, the three-dimensional structure of the multilayer is formed. Based on the above theories, Wu et al. researched a simple and reliable acoustic assembly method for rapid assembly of cell spheres in capillaries. They introduced coupled stationary sound waves which generated a linear pressure node array of 300 capture nodes to make it possible to continuously manufacture the sphere in a high-throughput way. The spherical cells of mouse embryonic carcinoma (P19) produced by this method grew well in vitro and maintained their differentiation potential, which demonstrated the feasibility of this method and further advanced the assembly of simple, high-throughput 3D spheres [82]. In addition, Chen et al. demonstrated the ability to manufacture multicellular spheres on a 3D acoustic platform. The fast and high-throughput properties of the method were proved by continuously manufacturing more than 150 controllable spheres. These spherical cells could be cultured for one week, which proved that the spherical aggregates made by this method have a good cell survival rate [77].

Using acoustic assembly, only a few seconds are needed to obtain simple geometry of microgel units. In addition to this, cell viability can also be highly maintained through this method. However, the aggregates formed by this technology are unstable, and secondary optical crosslinking appears to be particularly important. Secondly, when constructing the multilayer structure, the acoustic wave will also attenuate along the direction of gel thickness, so the final structure shape and layer number that can be formed have certain limitations.

### 3.2. Optical Tweezers

**Figure 9:**
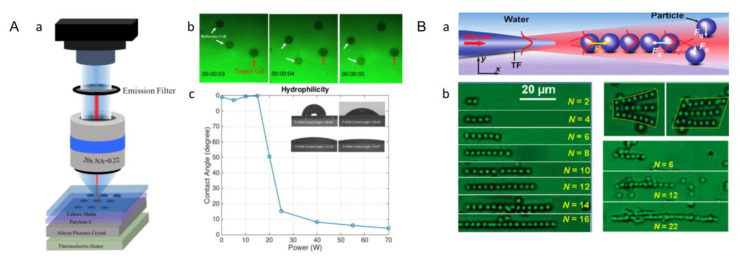
(**A**) Device diagram of optical tweezers. (Reproduced with permission from the Reference [80]). (**a**) The single-mode laser incident vertically on the photonic crystal, improving the capture efficiency through diffraction. The propen-c membrane on the substrate provides a biocompatible surface for cell culture and the culture temperature is controlled by a thermoelectric heater located below the substrate. (**b**) HPSCs (embryonic liver cells) are manipulated by using a propylene-assisted photonic crystal optical tweezers system. Manipulating cells’ movement on a hydrophobic polypropylene-C film by using a low-intensity laser beam with a 20× objective lens (N.A. = 0.22). Four cells are dragged into a rectangular pattern by optical tweezers, and the movement of one cell is indicated by the relative distance between the reference cell (white arrow) and the captured cell (red arrow), where the captured cells are located in the corner of the rectangular figure. (**c**) The relationship between plasma treatment power and hydrophilicity of propylene surface. Hydrophilicity is indicated by the contact angle of deionized water droplets on the surface. The relationship between the contact angle and the plasma power after 30 s of treatment with different plasma power. The plasma treatment power is 15 W, 20 W, 25 W, and 40 W, respectively. (**B**) Optical binding of yeast cell chains. (Reproduced with permission from the Reference [105]).

Optical tweezers use a highly focused laser beam used to capture and manipulate small particles in space with refractive indices different from those of the surrounding medium, which also known as a single-beam gradient force optical trap [104]. The optical tweezers method is a non-invasive tool widely used to operate nanoparticles, and has been widely used in tissue engineering because of its nontoxicity. When this light field interacts with the object, the whole object is affected by the light to achieve the effect of pinching, which can manipulate and capture particles from nanometer to micron scales. Traditional optical tweezers need high-intensity laser beams to generate enough light-trapping force, but the light damage limits the duration and application. To get over this disadvantage, methods to minimize photothermal damage and improve capture efficiency have been developed. This new technology inherits the versatility of traditional optical tweezers, and the efficiency of the capture force on the substrate of the photonic crystal is improved without affecting the cell viability. Previously, researchers manipulated hPSCs (embryonic liver cells) by using the photonic crystal optical tweezers system. A single-mode laser was inserted vertically into the photonic crystal and the embryonic hepatocyte movement was manipulated on the hydrophobic Parylene-C film. The laser light incident vertically on the photonic crystal creates an enhanced optical trap above the substrate, thus reducing the light damage to the cell (Figure 9A) [80]. At present, the single tapered fiber probe is also widely used to capture and manipulate particles or cells. Once a laser beam is fired into the tapered fiber probe (TFP), the light from the TFP after focusing exerts an optical force on the particles, which is formed by the gradient force (Fg) and the scattering force (Fs) (Figure 9B(a)). Fg tends to attract particles, while Fs tends to repel them, and both of them control the motion of particles together. Firstly, the scattering force will drive the first particle away along the TF axis. Subsequently, the particles beside the TF axis and near the first particle can be trapped to the TF axis resulting from transverse gradient force [105]. Under the action of the axial gradient force of the tail particle, the particle is closely bound to the former. Therefore, multiple particles are assembled together by the cooperation of the optical scattering force and the scattering force. This method can form chains of particles, or even two-dimensional particle arrays (Figure 9B(b)), and this binding ability can also act on cells in the same way, so the researchers have used this method to form chains of yeast cells. With the development of a single cone fiber optic probe, Kirkham et al. proposed a holographic technique based on optical tweezers to accurately generate customized honeycomb microstructures. Based on this method, hydrogel modules containing embryonic stem cells are assembled into different geometric shapes. Control of the microenvironment is achieved through the release of specific factors of the polymer particles in these structures to replicate structures in the adult stem cell niche. The application of micromanipulation based on holographic optical tweezers will enable researchers to form complex structures of cells, matrices and molecules with sub-micron precision, thus gaining new insights into the biological microenvironment [104].

Accuracy and reliability is the greatest advantage of optical tweezers, and it can also be used for single cell construction. However, in this way, cell activity is affected.

### 3.3. DNA-Assisted Assembly

Deoxyribonucleic acid (DNA) is one of the four biological macromolecules contained in biological cells. There are four kinds of bases: adenine (A), guanine (G), thymine (T) and cytosine (C). Adenine (A) and thymine (T) are linked in a double bond, and cytosine (C) and guanine (G) are linked in a triple bond. Thus, researchers have used this principle to assemble microgels through the DNA-assisted assembly method [94]. 

**Figure 10:**
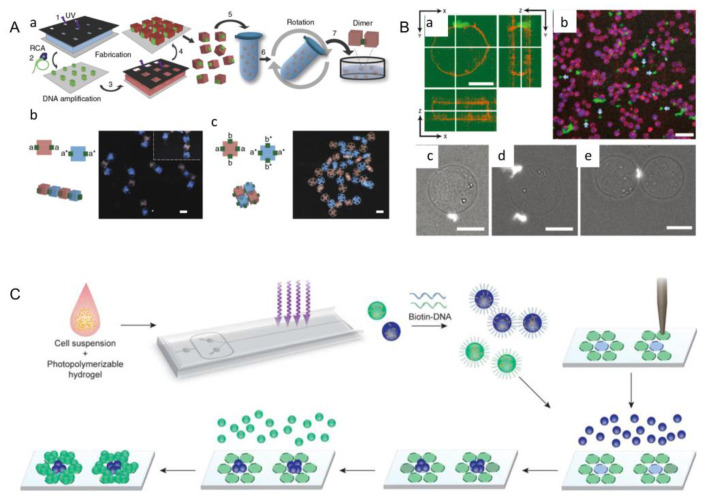
(**A**) The process of the DNA-assisted assembly. (Reproduced with permission from the Reference [95]). (**a**) The hydrogels that surround the cells are labeled with different single strands of DNA. According to the principle of base complementary pairing, the hydrogel dimer is formed under the action of hydrogen bond between corresponding bases. Four different DNA strands are used to decorate the four sides of the cubic hydrogel units, so that the specific connection could be realized in four different directions, and the chain and mesh assembly structures are finally obtained. (**B**) Attachment of fluorescent DNA hexagonal arrays through streptavidin (STV) to biotinylated Jurkat cells. (**a**) Confocal cross-section of fluorescent-labeled DNA-biotin-STV array binding to biotinylated Jurkat (leukemia) cells. Jurkat cells appear orange on the surface. (**b**) Confocal microscopy field of fluorescent-labeled DNA-biotin array binding to biotinylated Jurkat cells. Unbound arrays are represented by light blue arrows. The DNA array is green and the Jurkat cell surface is red; Jurkat’s cytoplasm is blue. (**c**–**e**) fluorescence micrograph DNA-biotin array binds to Jurkat-biotin cells. (Reproduced with permission from the Reference [94]). (**C**) Using DNA’s good molecular recognition ability to achieve rapid template assembly for multiple microtissue types. The cells are injected into a photopolymerizable hydrogel pre-polymer high flux microfluidic encapsulation device. Droplets of the cell-prepolymer mixture are exposed to ultraviolet light on the chip to form streptavidin-containing microstructures, which are then terminated with biotin oligonucleotides. Encoded microtissues containing different cell types are seeded onto a DNA microarray template, which guide the microtissues to bind to specific points on the surface of the template to obtain cell-bearing microtissues. (Reproduced with permission from the Reference [94]).

The DNA-assisted assembly method was first proposed by Qi et al. In this assembly process, cell-loaded hydrogels are marked by different single strands of DNA, which are specifically linked in pairs according to the principle of base complementary pairing, and hydrogel dimers are formed under the action of hydrogen bonds between corresponding bases (Figure 10A) [95]. In order to form a more complex structure, Qi and his team decorated the four faces of the cubic hydrogel unit with four different DNA single strands, enabling it to achieve specific connection in four different directions. Finally, they obtained the assembly structure of chain (Figure 10A(b)) and mesh (Figure 10A(c)). The strength of the DNA bond can be changed by adjusting the density and length of the DNA on the gel surface, in which long strands of DNA can be obtained by rolling loop amplification using a short DNA primer to optimize the assembly process.

In the DNA-assisted assembly method, Koyfman et al. demonstrated how self-assembled DNA arrays can be directed to the surface of cells. The first approach used streptavidin (STV) as a bridging component, in which biotin-modified arrays were combined with biotinylated cancer cells (Figure 10B) [94,97]. This approach involved the use of a non-specific pathway of lysine-reactive biotin. The second approach used STV and antibody as bridging elements to bind the biotin-modified array on the antibody to the natural epidermal growth factor receptor (EGFR) expressed on certain cancer cells. It built on the previous one, but used interactions between cell-specific surface proteins and their antibodies. In addition, the lipid-DNA hybridization system was studied by Xiao et al. After a brief introduction to the biophysical and biochemical properties of the membrane, they emphatically introduced the representative ways that DNA interacts with the membrane, including electrostatic interactions, membrane anchors, and membrane-bound proteins. Finally, they discussed the properties of DNA functionalized membranes and the factors for membrane-anchored DNA, and then reviewed the applications of lipid–DNA hybridization systems in recent years [96].

With the above theoretical basis, rapid assembly of various microtissue types has been realized. At first, cells were injected into a photopolymerizable hydrogel pre-polymer high-flux microfluidic encapsulation device. Then, the droplets of the cell-prepolymer mixture were exposed to ultraviolet light on the chip to form streptavidin-containing microstructures, which were then terminated with biotin oligonucleotides. Finally, the encoded microtissues containing different cell types were seeded onto a DNA microarray that guided the microtissues to bind to specific points on the surface of the template to obtain microtissues with cells (Figure 10C) [94]. Although the potential applications of DNA-assisted assembly in bottom-up tissue engineering have been proved, the potential of constructing three-dimensional complex tissue structure remains to be further explored.

### 3.4. Magnetic-Assisted Assembly

Magnetic-assisted assembly could be achieved in a non-uniform magnetic field, in which module units assemble under the force of the magnetic field [66]. Firstly, magnetic nanoparticles are added to the module element, and these particles can be directly labeled on the surface of cell membranes or microgels. Now, magnetic ferric oxide nanoparticles (Fe_3_O_4_) and cationic liposomes are widely used as magnetic nanoparticles. Then, driven by an external magnetic field, the labeled cells are oriented to gather into cell layers, which can be accumulated layer by layer to form a 3D structure.

**Figure 11:**
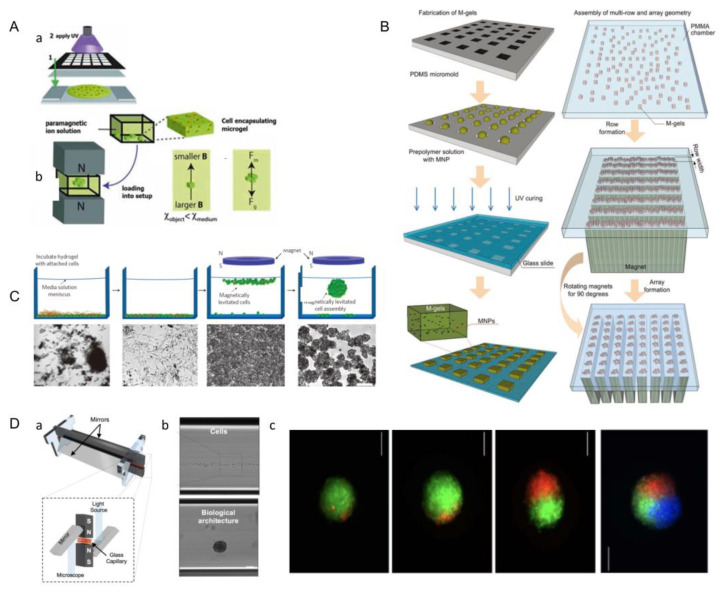
Schematic diagram of assembly guided by magnetic field. (**A**) The hydrogel units are fabricated using optical graphics with patterned masks. The hydrogel prepolymer solution is moved onto a slide and then exposed to ultraviolet light (**a**). In a magnetic device consisting of two opposite magnets with the same magnetic pole, the suspended cells are guided by magnetic forces to assemble into structural blocks (**b**). If the magnetic susceptibility of the object is lower than that of the suspended medium, the object moves from a higher magnetic field intensity to a lower magnetic field intensity at the center line between the two magnets. The forces acting on the suspended object at equilibrium height include magnetic force (Fm) and modified gravity (Fg), which is the difference value between gravity and buoyancy. (Reproduced with permission from the Reference [70].) (**B**) The cells labeled with magnetic nanoparticles are wrapped in microgel, and the microgels are assembled under the action of magnetic field, and then the macro-scale engineered tissue can be formed. (Reproduced with permission from the Reference [68]). (**C**) Magnetic levitation three-dimensional cell culture. The upper row is the schematic diagram of cell suspension, and the lower row is the corresponding optical micrograph of neural stem cells at each stage. The dark spots are remnants of hydrogel fragments. Wash to remove excess hydrogel fragments. After placing a magnet above the liquid level of a petri dish, cell-encased microgels are found to accumulate beneath the surface of the liquid. After suspension for 12 h, characteristic multicellular structures (single structure in the schematic diagram) are formed. (Reproduced with permission from the Reference [67]). (**D**) A biocompatible magnetic levitation assembly system. (**a**) This system consists of two magnets with a glass microcapillary between them and a mirror added on the side for imaging and real-time monitoring. (**b**) Aggregation of cells in a microcapillary under magnetic force. (**c**) Fuse single cells and spheres of different or similar sizes under magnetic guidance to obtain cellular structures with heterogeneous compositions.

The optical-based fabricating method can also be perfectly combined with this method to construct and assemble tissue modules. The photoresist with a patterned mask was used to fabricate cell-loaded hydrogels (Figure 11A(a)) [70]. Then, the cell-loaded hydrogel module units were placed between two opposite magnets of the same polarity (Figure 11A(b)). The forces acting on a suspended object at equilibrium height include magnetic force (Fm) and modified gravity (Fg), which is the difference between gravity and buoyancy. The cell-loaded microgels are assembled under the action of magnetic field, and then the macro-scale engineered tissue can be formed. To precisely create 3D cell structures, Tocchio et al. developed a biocompatible magnetic levitation assembly system. This system consists of two magnets with a glass microcapillary between them and a mirror added on the side for imaging and real-time monitoring (Figure 11D(a)). Living cells with intact membranes have different susceptibility to the surrounding medium, and can therefore be assembled in suspension, whereas dead cells and cell fragments sink to the bottom of capillaries and cannot be induced to assemble (Figure 11D(b)). This approach has a unique advantage over existing techniques, because dead cells and cell fragments can influence spherical features (such as shape and size), as well as cell behavior; however, these problems can be selectively removed by the technique. Following the invention, Tocchio et al. used this device to fuse single cells and spheres of different or similar sizes under magnetic guidance to obtain cellular structures with heterogeneous compositions (Figure 11D(c)) [68].

Xu et al. made microgel modules loaded with magnetic nanoparticles and cells. Through the spatial control of the magnetic field, the 3D structure could be manipulated to achieve multi-layer assembly of multiple microgel layers. Firstly, the cells were encapsulated in the hydrogel containing phage, magnetic iron oxide, and gold nanoparticles by the micromolding method, and then the cell-loaded microgel module units were prepared by optical crosslinking. Finally, the magnetic microgel modules were assembled by magnetic assemblers. During the process of magnetic assembly, parallel magnets separated by polymethyl methacrylate (PMMA) spacers aligned the randomly distributed microgels in a row. These module units were then assembled into an array by rotating magnets by 90 degrees to the base of the chamber. The magnetic assembler developed by Xu et al. has the potential to have a significant impact on tissue engineering, regenerative medicine, pharmacology, and stem cell research (Figure 11B) [68].

In a late study of magnetic assembly, Xu et al. offered to coat magnetic nanoparticle labeled cells in microgel, and then, engineering tissues at the macro-scale could be obtained by using a magnetic field. By controlling the distribution of magnetic fields, the different three-dimensional structures can be constructed [68]. At present, researchers have raised a new method called magnetic levitation, in which the permanent magnet is hung on the culture medium, the cells or gels labeled by magnetic particles are suspended on the surface of culture mediums and aggregate to form cell clusters under the action of the magnetic field (Figure 11C) [67]. Moving the magnet or changing the geometry of the magnet can modify the spatial gradient distribution of the magnetic field; thus, the three-dimensional structures of the aggregated cells or gel masses can be changed. 

This method is simple to operate and has high control precision, but the magnetic nanoparticles may affect the activity of cells, and the weakening of magnetic fields may also affect the results of assembly.

### 3.5. Surface Modification

#### 3.5.1. Thermosensitive Surface Hydrophobicity

**Figure 12:**
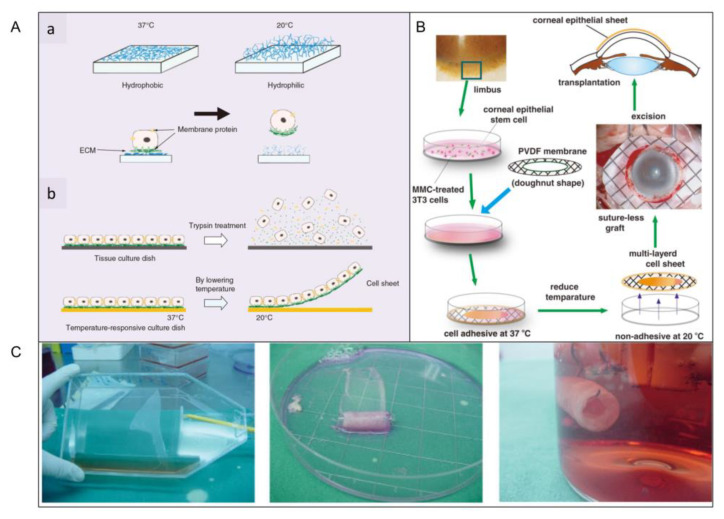
(**A**) Temperature-sensitive petri dishes are used to show attachment and separation of thin slices of cells. (**a**) At 37 °C, cells attach to the hydrophobic surface, and below 32 °C, cells detach from the hydrophilic surface. Below 32 °C, the poly (*N*-isopropylacrylamide) is rapidly hydrated and non-sticky because the surface feature has changed from hydrophobic to hydrophilic. Cells are connected to each other through intercellular junctions and ECM. (**b**) When enzymes are introduced to remove cells from the surface, the connections between these cells and the ECM are seriously broken. When cultured on a thermosensitive surface, the connections between cells can be well preserved. (Reproduced with permission from the Reference [64]). (**B**) Limbal stem cells are collected and planted on a temperature-sensitive culture surface. After 2 weeks of culture in the mitomycin-treated 3T3 feeder layer, layers of cells can be separated from the temperature-sensitive surface by reducing the temperature. Then, the cells are harvested by using doughnut-shaped polyvinylidene fluoride (PVDF) membranes as thin sheets. (Reproduced with permission from the Reference [56]). (**C**) Thin slices of cells are extracted from continuous culture of bone marrow-derived cells. Thin slices of cultured bone marrow-derived progenitor cells are removed from the temperature-sensitive surface by reducing the temperature. Wrapping the cell sheet on a cylindrical PLGA mesh. (Reproduced with permission from the Reference [52]).

With the development of bottom-up tissue engineering, researchers have developed a method to peel off cell sheets from thermosensitive surfaces [56,59]. Today, thermosensitive surfaces have been skillfully used in the biomedical field to prepare liver-like microtissues, urothelial tissues, epicardium, blood vessels, and corneal tissues. When the culture temperature is higher than the minimum critical temperature (32 °C), the surface is hydrophobic. When the temperature drops below 32 °C, the surface reversibly changes to hydrophilic rather than cellular adhesion due to the rapid hydration and expansion of the graft (Figure 12A(a)) [64]. In contrast to enzymatic hydrolysis, only the adhesive proteins interact with the material surface are released and the cells with intact membrane proteins and adhesive proteins are dropped from thermosensitive surface (Figure 12A(b)). 

Nishida et al. used this method to obtain the corneal tissue in vitro. Limbal stem cells were collected and planted on a temperature-sensitive culture surface. After 2 weeks of culture in a 3T3 feeder treated with mitomycin-C (MMC), all multilayer cells were released by cooling to obtain cell sheets, which could be transplanted to the corneal stroma without suturing (Figure 12B) [56]. Sheets of bone cells and cardiomyocytes can be made in the same way (Figure 12C) [52]. Poly(*N*-isopropylacrylamide) is a kind of temperature-responsive material, which is frequently used in this technology. Yamada et al. cultured bovine liver cells in a polystyrene dish with Poly(*N*-isopropylacrylamide) grafted on the surface, and observed whether the cells were detached from the surface of dish by changing the culture temperature. In this study, it was found that the grafted surface of thermally responsive polymers could be used as a temperature switch to control cell culture and isolation, and successive subculture of bovine liver cells could be achieved without the influence of enzymes on cell activity [52]. In addition, Yamato et al. also used this method to harvest a human keratinocytes sheet. They placed human keratinocytes in the petri dish grafted with a heat-responsive polymer called *N*-isopropylacrylamide. Keratinocytes proliferated on the surface of the petri dish at 37 °C and formed a multilayer keratinocytes sheet. Subsequently, the multilayer keratinocytes could be separated from the graft surface by lowering the temperature to 20 °C to obtain a complete keratinocytes sheet [120].

Cellular activity in 3D structures fabricated by temperature-sensitive surfaces is well-protected. However, cell sheets made by this way are two-dimensional, lacking three-dimensional complex tissue. Meanwhile, the structure has insufficient controllability and low spatial resolution.

#### 3.5.2. Directional Assembly on Hydrophilic and Hydrophobic Surfaces

**Figure 13:**
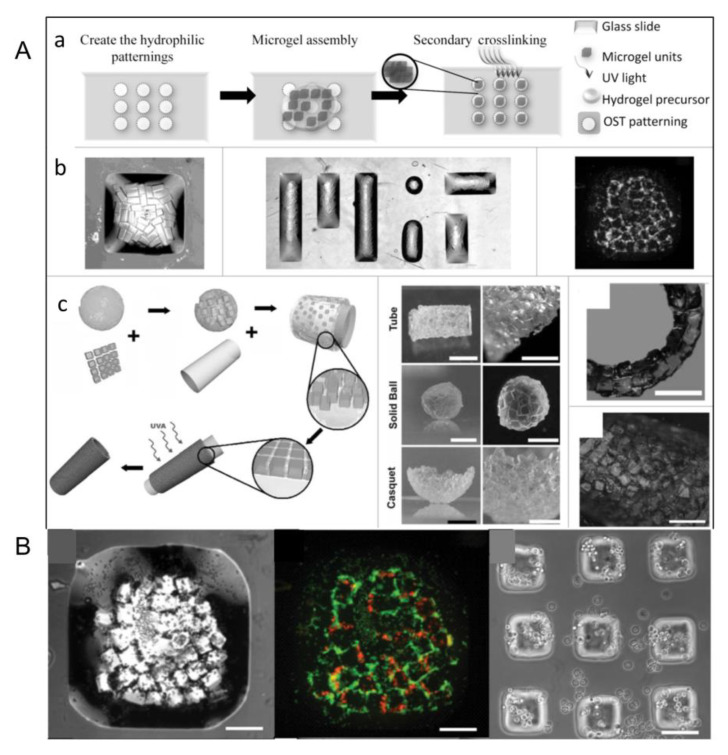
(**A**) Directional assembly of cell-loaded microgels in water droplets on surfaces with hydrophobic and hydrophilic regions. (**a**) Driven by surface tension, hydrophilic microgels aggregate in patterned water droplets that are confined within the hydrophilic pattern and stabilized by secondary cross-linking. (**b**) By controlling the size and shape of the surface pattern, well-structured microgel assemblies are formed on the glass slide. Stably assembled microgels can be easily obtained from the surface, and finally, multicellular tissue structures can be obtained. (**c**) To make microgel assemblies with complex shapes, microgels of a specific shape are first mixed in a prepolymer solution and deposited on the surface of PDMS treated with oxygen plasma. Due to hydrophilicity, the liquid wetted the surface and drove the microgel subunit to cover the PDMS surface. After the excess prepolymer solution is removed, microgels are assembled into tightly packed sheets on the surface of the template (bottom-right corner). Complex-shaped microgel components, such as tubes, spheres, and shells (right). A further application of the method has also shown the construction of larger structure in a layer-by-layer manner, in which double-layer tubes with diameters of 5 mm are made. Hepatocytes (HepG2) are encapsulated with PEG microgel and assembled into tubular structures. (Reproduced with permission from the Reference [25]). (**B**) Cell-loaded microgels are assembled by using block hydrogels of specific shape. The morphology and viability of the cell-loaded microgels are stained before assembly and 24 h after culture. (Reproduced with permission from the Reference [59]).

The cell-loaded microgels can achieve directional assembly in water droplets with hydrophilic and hydrophobic surfaces [53], which can be stabilized by secondary cross-linking (Figure 13A(a)) [59]. Based on the above properties, by controlling the size and shape of the surface pattern, well-structured microgel assembly can be formed on the glass slide (Figure 13A(b)) and microgel components with complex shapes, such as tubes, spheres, and shells, can also be formed through this method (Figure 13A(c)). Using this technique, Javier et al. constructed microgel module unit aggregates with various structures using polydimethylsiloxane (PDMS) as an assembly template. They created hollow tubular structures using PDMS columns as templates for microgel assembly, and the hemispherical enclosures were obtained in the same way. The spherical structure is also an example of self-aggregation of hydrophilic blocks in hydrophobic media. However, unlike the preceding two examples, it was made without the mold. When microgel module units are added to a low-density hydrophobic medium (such as mineral oil), they spontaneously assemble into a compact sphere. Cell activity is still an important indicator of whether a method is feasible, so after obtaining the ideal structure, we need to test the activity of the cells in it. Cells dyed red represent dead cells, and cells dyed green represent living cells [59]. Results show that the cell-loaded microgel can be assembled into predefined geometric and biological characteristics of cells that remained highly active (Figure 13B) [65]. However, in the flat-surface mode, the resulting microgel components are limited to two-dimensional (2D) structures and are suitable for simulating simple tissues (such as skin).

#### 3.5.3. Interface Self-Assembly

**Figure 14:**
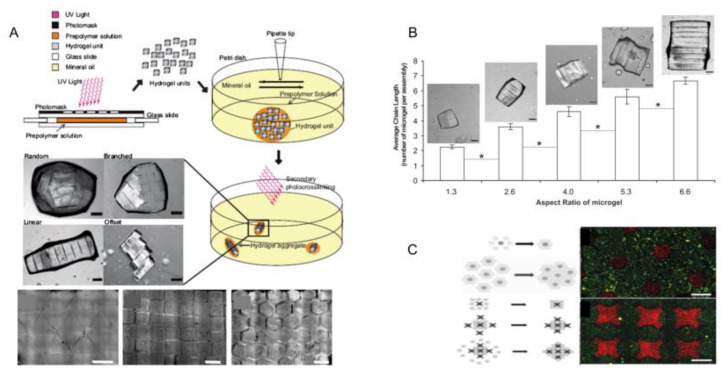
(**A**) Hydrogel modules are created directly through masks by UV photopolymerization, then hydrogel modules are polymerized and self-assembled in a hydrophobic medium (mineral oil). The second optical crosslinking solidifies the structure, which varies in size and structure according to the geometry of the module. (Reproduced with permission from the Reference [1]). (**B**) Effect of microgel size on microgel assembly. The average chain lengths of linear, branched or random microgel combinations containing microgel units with different aspect ratios are compared. (Reproduced with permission from the Reference [58]). (**C**) Schematic diagram of microgels assembling complex building blocks on PFDC or CCL_4_ surfaces. One cell type (dark gray) is surrounded by a second cell type (light gray). A complex building-block assembly schematic using lock-and-key shaped microgels to better guide the assembly process for controlled coculture. (Reproduced with permission from the Reference [60]).

Module units can aggregate at the interface of two different media driven by surface tension to form ordered two-dimensional or three-dimensional structures [65]. Hydrogel modules were placed in a hydrophobic medium (mineral oil), and tissue with controllable three-dimensional structure can be achieved by stirring the solution under surface tension (Figure 14A) [1]. Now, interface self-assembly has been divided into two parts: Liquid–liquid interface self-assembly and liquid–gas interface self-assembly. In the liquid–liquid self-assembly system based on oil–water two-phase flow, hydrophobic application is the main driving force of self-assembly. PFDC and CCL_4_ are selected from a range of possible solutions because they are denser than water, ensuring that the microgel blocks will float. However, due to the excessive toxicity of CCL_4_, only PFDC is used in cell-loading and -assembly experiments. Under the action of the thermodynamic trend, the module units aggregate and eventually assemble into a structure with minimal surface free energy, whose shape may be influenced by experimental parameters, such as stirring speed, stirring time, aspect ratio of the. module unit, and so on. Additionally, the length of the assembled chain structure is related to the horizontal and vertical ratio of the microgel module (Figure 14B) [58]. In a later study, Zamanian et al. obtained cocultured microgel structures based on this method. Firstly, the microgel modules loaded with a kind of cell were placed on the surface of PFDC. Then, these modules were surrounded by other microgel modules loaded with another kind of cell. Finally, the aggregation of microgel modules with complex structures was obtained by surface tension (Figure 14C) [60].

The potential superiority of this approach is repeatedly achieved by organizational structures without complex assembly and operation procedures. However, complex three-dimensional structures cannot be fabricated by this method, and the problem of random or uncontrolled structures still remains. 

### 3.6. Robots-Assistant Assembly 

**Figure 15:**
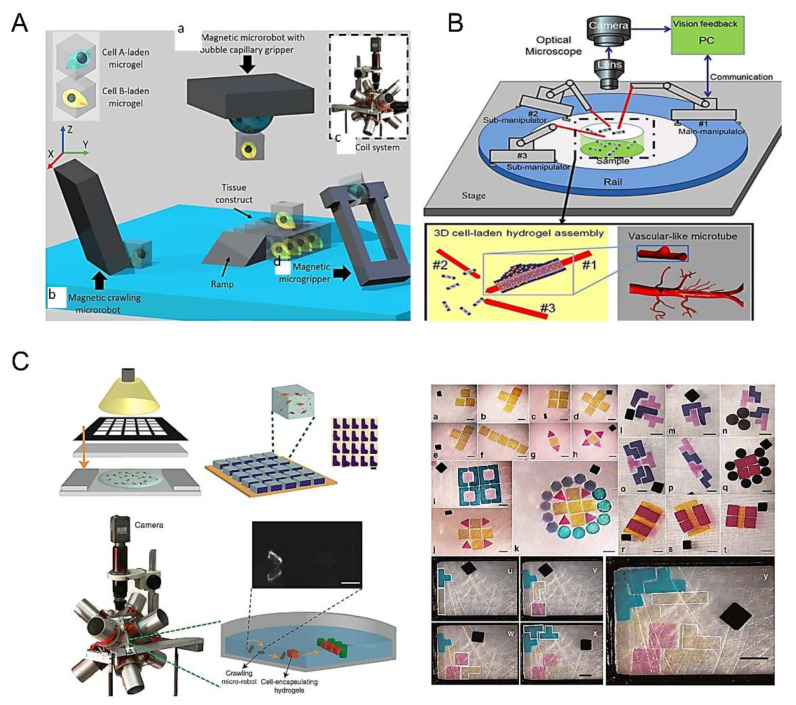
(**A**) Three-dimensional assembly of cell microgels carried by different microrobots. (**a**) Magnetic microrobot with bubble capillary gripper. By changing the pressure in the working environment, bubbles can be stretched and contracted to pick up and release microobjects. (**b**) Magnetic crawling micro-robots. Microgels are pushed to the desired position by the microrobot, and a micromachined ramp is used to lift the microbots higher up the organizational structure. (**c**) Magnetic coil system for microrobot control. (**d**) Magnetic microforceps. The jaw picks up and releases microobjects by turning an external magnetic field on and off. By changing the pressure in the working environment, bubbles can be stretched and contracted to pick up and release microobjects. (Reproduced with permission from the Reference [101]). (**B**) Robot-assisted assembly is used to assemble blood vessels. (Reproduced with permission from the Reference [100]). (**C**) Assembling micromodules by using magnetic robots. (I) Preparation of cell potting hydrogel by UV crosslinking; (II) Micrographs of hydrogel preparation and L-type hydrogel preparation; (III) Magnetic coil system for remote control of magnetic micro-robots; (IV) Alignment of unconstrained magnetic micro-robot movement with building units. Alignment of two-dimensional microrobots in hydrogel configuration (right). Microrobots are used to arrange and reconstruct polyethylene glycol dimethacrylate hydrogels (I–XI) and methacrylate gelatin hydrogels (XII–XX) of different shapes to form complex plane structure. The black object in each image is a top view of a crawling micro-robot. (Reproduced with permission from the Reference [102]).

In recent years, microrobot technology has been developed to observe, measure and fix nanoscale objects. In particular, the combination of microgel assembly technology and an optical or magnetic microrobot system provides a new approach for bottom-up tissue engineering [99]. There are four types of microbots in common use (Figure 15A): (a) Magnetic microrobot with bubble capillary gripper. By changing the pressure in the working environment, bubbles can be stretched and contracted to pick up and release microobjects [121,122]. (b) Magnetic crawling micro-robots. Microgels are pushed to the desired position by the microrobot, and a micromachined ramp is used to lift the microbots higher up the organizational structure. (c) Magnetic coil system for microrobot control [123]. (d) Magnetic microforceps. The jaw picks up and releases microobjects by turning an external magnetic field on and off. By changing the pressure in the working environment, bubbles can be stretched and contracted to pick up and release microobjects. Among these four kinds of microrobots, the magnetic coil microrobot has been most widely used [101]. Tasoglu et al. assembled cell-loaded microgel modules into complex 3D structures by remote magnetic field control of cordless magnetic microrobots (Figure 15C) [99]. Firstly, cell-loaded microgel modules were prepared by UV-light crosslinking. The shape of the microgel module was determined by the mesh size in the mask and the thickness of the spacer layer. Hydrogel precursors were exposed to ultraviolet light to prepare microgel modules of different shapes and placed them in phosphate-buffered brine. The magnetic microrobot, consisting of NdFeB particles wrapped in a polyurethane adhesive, was placed on the bottom of the assembly chamber and driven by a system of eight electromagnets surrounding the workspace. The microgel modules were propelled and assembled by algorithmically controlled microrobots whose magnetic fields can be dynamically adjusted in response to user input. The robot’s movement is guided by a magnetic force of varying sizes, and small oscillating magnetic torques are used to break down surface friction, which is usually the main cause of such micro-contacts.

In tissue manufacturing, microrobots can be used to assemble blood vessels (Figure 15B) [99]. In this process, the microrobot uses the pickup and placement function to thread the microgel module units onto the robotic arm and eventually form blood vessels [102]. The arm is divided into the double manipulator transition micromodule, double manipulator non-transition micromodule, and single manipulator up-and-down microassembly [100].

Assembly of heterogeneous materials with high precision is the biggest advantage of robotic-based assembly. However, this method also has the disadvantage of low throughput. Additionally, flexibility, power, and positioning remain major challenges, greatly limiting the application of viable robot design from in vitro to preclinical stages. Overall, the cordless mobile micro-robots currently open up new avenues for biomedical applications, and pave the way for minimally invasive and cost-effective strategies that lead to rapid patient recovery and an improved quality of life.

### 3.7. Dielectrophoresis Method

In recent years, great progress has been made in the studies of microscale operations and analysis of biological cells [83]. Microelectromechanical systems are also increasingly being used to selectively capture, manipulate, and separate micro/nano biological objects. The term “dielectrophoresis” was coined by Herbert in 1951 [86]. He defined it as “the motion of a suspended particle with respect to a solvent caused by the polarizing force of an uneven electric field”. Additionally, the meso-electrophoretic forces acting on particles have a gradient of electric field intensity squared [87,88,89,91,93].

(1) $$F_{DEP}=2\pi \varepsilon_{m}r^{3}Re\left[CM\right]\nabla {\left|E\right|}^{2}$$

(2) $$Re\left(CM\right)=Re\left(\frac{\tilde{\varepsilon_{p}}-\tilde{\varepsilon_{m}}}{\tilde{\varepsilon_{p}}+2\tilde{\varepsilon_{m}}}\right)$$

(3) $$\;\tilde{\varepsilon }\;=\varepsilon -j\frac{\sigma }{\omega }$$

(4) $$\tilde{\varepsilon_{p}}=\frac{\left(C_{memb}+\frac{G_{memb}}{j\omega }\right)r\times \left(\varepsilon_{o}\varepsilon_{r_{c}}+\frac{\sigma_{c}}{j\omega }\right)}{\left(C_{memb}+\frac{G_{memb}}{j\omega }\right)r+\left(\varepsilon_{o}\varepsilon_{r_{c}}+\frac{\sigma_{c}}{j\omega }\right)}$$

$\varepsilon_{m}—\mathrm{dielectric}\;\mathrm{constant}$;

$\tilde{\varepsilon_{p}}—\mathrm{complex}\;\mathrm{permittivity}$;

$\tilde{\varepsilon_{m}}—\mathrm{Nonuniform}\;\mathrm{permittivity}$;

$\sigma_{c}—\mathrm{conductance}$;

$C_{memb},{\;G}_{memb}—\mathrm{dielectric}\;\mathrm{properties}$.

**Figure 16:**
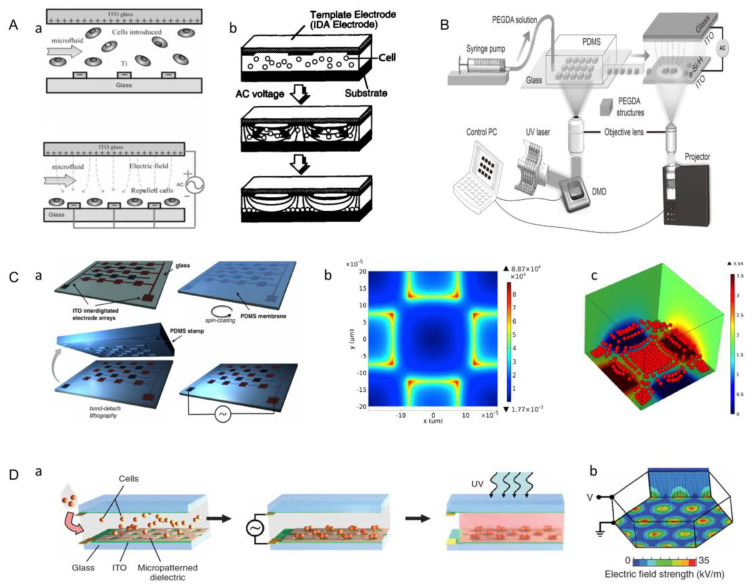
(**A**) Working principle of liver cell graphics chip. (**a**) A side view of a spatially randomly distributed cell (up) and side view of cell distribution under n-DEP. Hepatocytes are injected into the microfluidic cavity with continuous flow input and randomly distributed along the flow direction. (**b**) When sufficient alternating current (AC) is applied, the randomly distributed cells are repelled by the n-DEP effect under the control of the balancing force generated by DEP and fluid dynamics, and are arranged between the electrodes into the desired bead array pattern (Reproduced with permission from the Reference [84]). (**B**) Schematic illustration of the high-throughput fabrication and flexible manipulation system. The system consists of two main components: a DMD-based hydrogel manufacturing system for on-demand manufacturing; an ODEP force-based operation and assembly system for precise control. (Reproduced with permission from the Reference [45]). (**C**) (**a**) Schematic diagram of a quadrupole electrode array device. (**b**) Distribution of electric field intensity. (**c**) The aggregation of cells. (Reproduced with permission from the Reference [125]). (**D**) (**a**) The photosensitive prepolymer solution containing cells was filled between two conductive indium tin oxide (ITO)-coated plates. An insulating photoepoxy resin (SU-8) mask was placed on the bottom electrode plate. The insulating area covered most of the conductive surface, forming electrodes in the non-insulating area. An AC voltage was applied to the top and bottom plates, producing a spatially non-uniform electric field. (**b**) Distribution diagram of electric field intensity. (Reproduced with permission from the Reference [126]).

When the particle is more polarizable than the immersed medium, the resulting force directs the particle to the region of the maximum electric field. This phenomenon is called positive DEP (p-DEP) [83]. In contrast, the degree of polarization of the cell is lower than that of the suspended medium in an inhomogeneous electric field, which describes the movement of the particle away from the high field region, and this phenomenon is called negative DEP (n-DEP) [84]. Negative dielectric electrophoresis (n-DEP) cell manipulation is an effective method to simulate human hepatocytes in microelectrode arrays, and cell viability maintenance is an important objective of this method [124]. Puttaswamy et al. investigated the effects of low-conductivity media and optimally designed microchips on cell viability and cell adhesion [84]. Firstly, a glass substrate was used to fabricate a chip with titanium electrodes, which were arranged in parallel and radiated outward from the center. Hepatocytes were then injected into the microfluidic chamber, showing random distribution of cells (Figure 16A(a)). When sufficient alternating current (AC) was applied, the randomly distributed cells were repelled by the n-DEP effect under the control of the balancing force generated by DEP and fluid dynamics, and were arranged between the electrodes into the desired bead array pattern (Figure 16A(b)). This radial pattern simulated the lobule morphology of real liver tissue. Then, the activity of liver cells was tested and cell adhesion was quantitatively analyzed by a microfluidic shearing device. Finally, it was proved that the use of a low-conductivity medium, n-DEP mode, and optimized microchip resulted in significantly increased cell viability and cell adhesion rates.

Additionally, using negative dielectric electrophoresis to manipulate cells to achieve assembly, Menad et al. designed a quadrupole electrode array consisting of coplanar electrodes partially covered with a thin micropatterned PDMS film [125]. Thin PDMS layers were coated with poly-L-lysine, which acted as adhesion cells (Figure 16C(a)). The distribution of electric field intensity after the electrode was energized, is shown in the (Figure 16C(b)). The chip used the repulsion by negative-dielectric electrophoresis to assemble living cells into compact aggregates, or to attach living cell patterns to polylysine-functionalized PDMs surfaces (Figure 16C(c)). This method of cell aggregation is suitable for almost all kinds of animal cells [91].

Later researchers combined optical-induced dielectrophoresis (ODEP) with the digital micromirror device (DMD) for bottom-up tissue construction (Figure 16B) [45]. The system consists of two main components: a DMD-based hydrogel manufacturing system to produce the desired shape of the microgel module unit; and ODEP to provide a dielectric electrophoretic force to guide the assembly of modular units. The digital micromirror device (DMD) is used as a light modulator to polymerize arbitrary hydrogel microstructures in a high-throughput and on-demand manner. Many different microstructures are transferred onto a photoinduced electrodynamics (OEK) chip, allowing the assembly of building blocks composed of functional components and the creation of a complex, reconfigurable, three-dimensional structure. 

In addition, Albrecht et al. combined the dielectrophoresis technique with photolithography to obtain 3D multicellular aggregates [126]. The photosensitive prepolymer solution containing cells was filled between two conductive indium tin oxide (ITO)-coated plates. An insulating photoepoxy resin (SU-8) mask was placed on the bottom electrode plate. The insulating area covered most of the conductive surface, forming electrodes in the non-insulating area. An AC voltage was applied to the top and bottom plates, producing a spatially non-uniform electric field (Figure 16D(a)). The electrophoretic force generated by an electric field made cells move to positions with high electric field intensity, and cell movement was completed in 1–3 min (Figure 16D(b)). After the cells were located, the hydrogel in areas not covered by the insulation mask was illuminated with UV light to solidify into a 3D multicellular aggregate, and the whole process took only 20 min. In short, assembling modular units in this way is fast, but may cause some damage to cells.

### 3.8. Microfluidic-Based Assembly 

**Figure 17:**
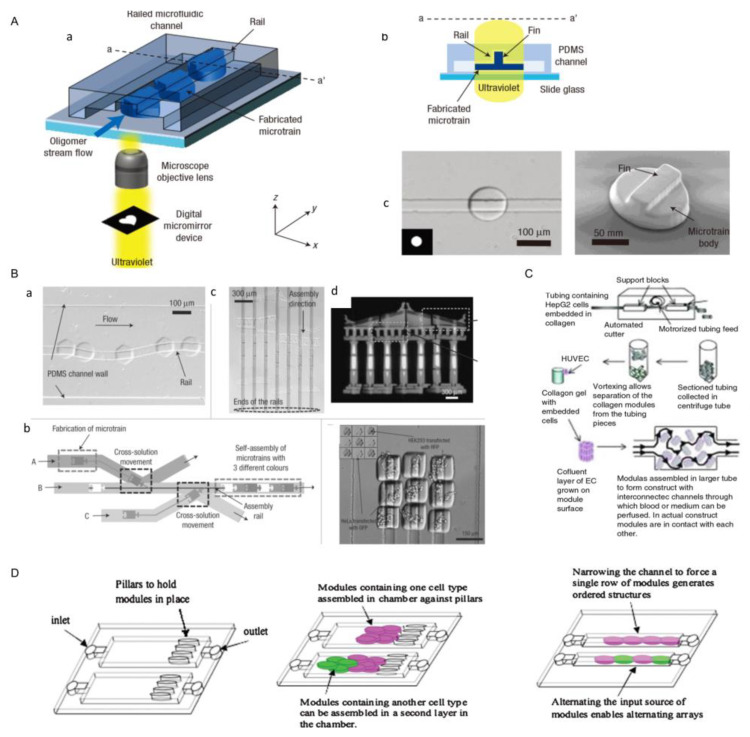
(**A**) (**a**) Schematic diagram of guide way microfluidic channel. (**b**,**c**) A fin-like cross-section of a guide way microfluidic channel. (Reproduced with permission from the Reference [130]). (**B**) (**a**) The guided motion of the micro-module follows a sinusoidal orbit. Each microstructure (37 microstructures in total) is made independently on the corresponding guide rail. (**b**) The heterogeneous assembly process based on the cross-solution motion scheme. The bottom right figure uses a cell building block containing PEG-DA hydrogel to assemble a 3 × 3 matrix from two different kinds of living cells. (Reproduced with permission from the Reference [25]). (**C**) Schematic of vessel assembly using microfluidic technology. (Reproduced with permission from the Reference [11]). (**D**) Assembly diagram of microfluidic chip module. Modules are squeezed into narrow microchannels to form ordered arrays. (Reproduced with permission from the Reference [109]).

One of the important characteristics of microfluidic is its unique fluid properties, such as laminar flow and droplets in a microscale environment. With the help of these unique fluid phenomena, microfluidic can achieve a series of micromachining and micromanipulation. Now, microfluidic is considered to have great development potential and wide application prospects in biomedical research [25,98,109,110,111,112]. With respect to in vitro bionics, microfluidic chips are very suitable for realizing physiological functions at the level of tissues and organs in vitro by using biomaterials such as bionic microstructures and hydrogels, which are also called “organ-on-chips” [113,114,115]. A fully deterministic method for guiding and assembling microstructures in fluid channels was proposed (Figure 17A(a)) [98]. In this device, the top surface of the PDMS channel guided the movement of the microstructures (Figure 17A(b)), which were manufactured with complementary polymer microstructures to fit the grooves (Figure 17A(c)). Complex one-dimensional and two-dimensional microstructures were constructed by using orbits as guiding mechanisms, and each microsystem consists of more than 30 hydrogel-based microstructures (each size less than 50 μm) (Figure 17B(a–d)). In addition, they achieved assembly of two hydrogel building blocks loaded with living cells. In another study, Peak et al. created microvascularized tissue by assembling tiny collagen rods [127]. In this approach, they constructed a collagen gel containing HepG2 cells using an automatic cutter, and fusion cell layers were obtained on the surface of the collagen components. The assembled modules were then injected with culture medium or whole blood and assisted by the gap between the collagen modules (Figure 17C) [11,127,128,129]. 

Bruzewicz et al. combined microfluidics technology with photolithography to develop a device for 3D culture of mammalian cells in microchannels (Figure 17D) [109]. A natural extracellular matrix, such as collagen, formed the matrix of each cell-loaded microgel module. In these modules, the flow of oxygen, nutrients, and metabolites in and out of the module was sufficient to allow the cells in the module to proliferate to the density similar to native tissue. Modules were loosely placed in the microfluidic channels and chambers to produce structures with a network of holes, through which cell culture could flow into the encapsulated cells. Within 24 h, the medium flowed through microfluidic channels and chambers at a constant rate. It was found that more than 99% of the cells in the microflow chamber were alive during this period of time.

## 4. Application

### 4.1. Tissue Engineering

Millions of patients are suffering from tissue damage every year [9,10,112,114]. Although tissue transplantation can be used to treat these patients, its use is limited by a severe shortage of donor tissue. However, tissue engineering can fill some of the gaps. At present, several scaffold-free, bottom-up tissue engineering approaches have been investigated to generate cartilage, bone and osteochondral structures, including self-assembly processes, granular cultures, and aggregative cultures.

#### 4.1.1. Bone Tissue

**Figure 18:**
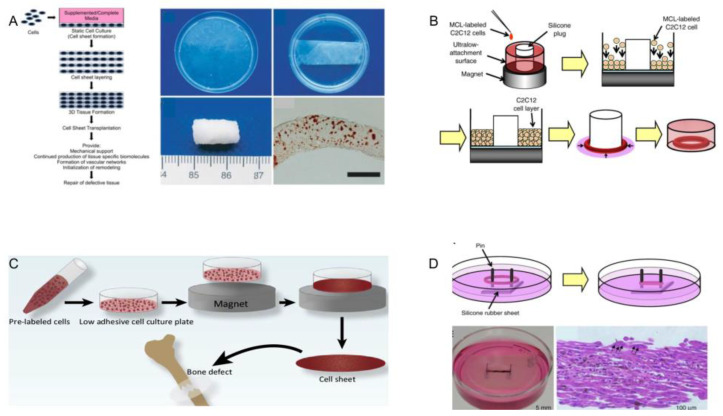
(**A**) Bone marrow mesenchymal stem cells (BMSCS) are cultured in osteogenic medium to form three-dimensional cell sheets. Histological examination (alizarin red staining) of the preimplantation tablet reveals mineralization. (Reproduced with permission from the Reference [131]). (**B**) Production of cell sheets with magnetically prelabeled cells. In a low-adhesion cell culture dish placed on a magnet, magnetically labeled cells undergo cell expansion in the dish to produce thin sheets of cells that are easy to separate. (Reproduced with permission from the Reference [132]). (**C**) Cell sheets are used for bone tissue repair. (Reproduced with permission from the Reference [69]). (**D**) Myocardium cell rings are constructed by magnetic assembly technology. A silicone plug is placed on an ultra-low adhesion surface to create a ring structure, and myocardium cells that marked by magnetic particles are implanted into a hole above the magnet. Cells are attracted by a magnetic force and accumulate on the surface of the culture. During 2D culture, the cell sheet contracts sharply, and the cells form a ring structure around the silica plug. A bright-field photograph of a honeycomb ring is shown, along with a bright-field micrograph of a cross-section stained with hematoxylin and eosin. (Reproduced with permission from the Reference [132]).

Osteoarthritis is a progressive chronic disease. Clinically, osteoarthritis manifests as a loss of synovial articular surface, which often results in discomfort, chronic pain, and reduces arthrosis movement. So far, there is no effective treatment for osteoarthritis [131]. Therefore, the development of tissue engineering (TE) for the treatment of osteoarthritis has attracted much attention. Castro et al. cultured bone marrow mesenchymal stem cells (BMSCS) in osteogenic medium to form three-dimensional cell sheets (Figure 18A). The cell sheet could be peeled away from the temperature-sensitive surface and used to repair the damaged bone tissue. The result shows that bottom-up tissue engineering can be used to repair damaged bone tissue.

Skeletal muscle is a type of striated muscles that attaches to bones, which is used for locomotion, posture, protection, heat production and fluid pumping. Replacement of muscle tissue is necessary after the loss of functions due to traumatic injury, tumor ablation, or muscle diseases such as muscular dystrophy. Yamamoto et al. used the method of magnetically assisted assembly to repair the damaged skeletal muscle. Firstly, the cells labeled with magnetite cationic liposomes were placed in a low-adhesion cell culture dish on a magnet. Additionally, they were magnetically attracted to the bottom of the plate, forming a dense sheet structure [132]. Finally, the magnetic labeled cells were expanded in the cell culture dish to produce a thin ring of cells (Figure 18B). Taking out the tissue ring and using it to cover the two columns, cell morphology and growth direction can be observed under a microscope, which also proves the feasibility of bottom-up tissue engineering to repair skeletal muscle [69].

#### 4.1.2. Cartilage Tissue

**Figure 19:**
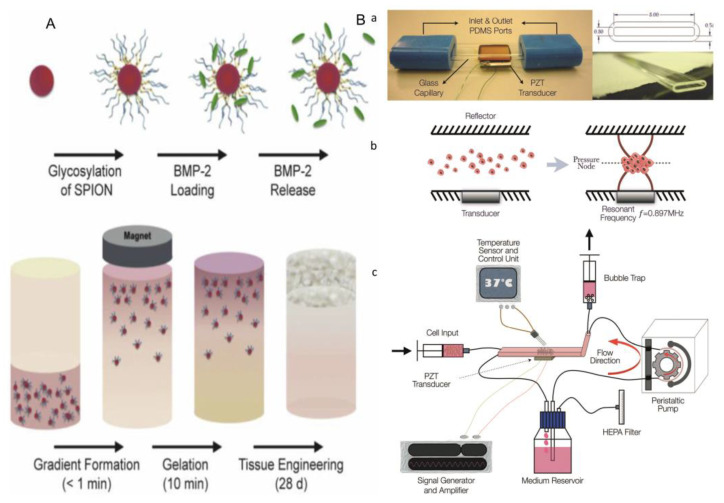
(**A**) Agarose hydrogels loaded with human bone marrow mesenchymal stem cells (HMSC) are heated and cultured for 28 days to produce rigid osteochondral structures composed of bone and cartilage tissue by arranging glycosyl spions (superparamagnetic iron oxide nanoparticles) fields by using an external magnetic field. (Reproduced with permission from the Reference [133]). (**B**) Application of acoustic fluid-controlled perfusion bioreactor in the transplantation of new cartilage. The bioreactor is prepared by rectangular glass capillary, polydimethylsiloxane (PDMS) connector and ceramic piezoelectric sensor (PZT). Schematic diagram of the formation of multicellular condensation in a resonator. The transducer produces an ultrasonic standing wave field in the capillary cavity, and the glass surface above it acts as a reflector. When cells suspended in the culture medium are introduced into the chamber, acoustic radiation forces direct the cells to the pressure nodes, in which they can rapidly aggregate and eventually form multicellular clumps that are suspended in the chamber above the transducer. The closed loop includes a resonant cavity, a bubble trap, and a syringe pump for introducing cells into the cavity. Wires welded to the sensor electrodes are connected to a custom amplifier driven by a signal generator. Although ultrasonic activation causes heating, at the ambient temperature of the system (36 °C), the active region of the chamber is stable at 37.0, plus or minus 0.5 °C. (Reproduced with permission from the Reference [134]).

As an autoimmune disease, rheumatoid arthritis causes damage to articular cartilage in the articular cavity. At present, the main clinical measures to repair articular cartilage include microfracture, autologous osteochondral transplantation, allogeneic osteochondral transplantation, and so on. Researchers have developed bottom-up tissue engineering to repair articular cartilage, which can effectively avoid many disadvantages in top-down tissue engineering [133]. In the construction of cartilage by magnetic assembly, spion binds to heparin to produce glycosylated crowns that effectively sequester and release growth factors. An external magnetic field is used to field-align glycosylated spions in a hMSC-laden agarose hydrogel, which is thermally gelled and cultured for 28 days to generate robust osteochondral constructs comprising both bone and cartilage tissue (Figure 19A). In addition, the acoustic fluid-controlled perfusion bioreactor has been widely used in cartilage transplantation. In this device, the transducer produces ultrasonic standing wave field in the capillary cavity, and the glass surface above it acts as reflector (Figure 19B(a)) [134]. When cells suspend in the culture medium are introduced into the chamber, acoustic radiation forces direct the cells to pressure nodes, in which they rapidly aggregate and eventually form multicellular clumps that suspend in the chamber above the transducer (Figure 19B(b)). The closed loop includes a resonant cavity, a bubble trap, and a syringe pump for introducing cells into the cavity (Figure 19B(c)). Although ultrasonic activation causes heating, at the ambient temperature of the system (36 °C), the active region of the chamber is stable at 37.0 ± 0.5 °C. The above two methods have proved the feasibility of bottom-up tissue engineering for cartilage repair. 

#### 4.1.3. Corneal Epithelial Tissue

**Figure 20:**
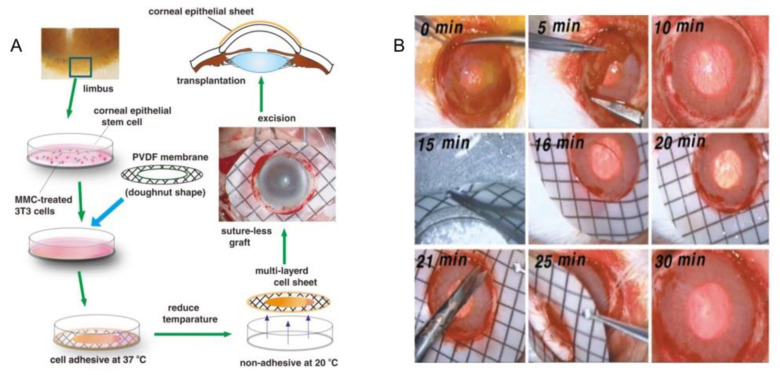
(**A**) Using a thermosensitive surface to obtain thin limbal stem cell sheets. After 2 weeks of culture in mitomycin-treated 3T3 feeding layers, cell sheets are stripped from temperature-sensitive surfaces by reducing temperature, and harvested by using donut-shaped polyvinylidene fluoride (PVDF) membranes. (**B**) Autologous transplantation of rabbit corneal epithelial cells. Continuous photographs of the migration process are shown. The label indicates the different time phases of the migration. Three weeks after keratectomy, neovascularization occurred all over the corneal surface. Hematoxylin-eosin staining is used 3 weeks postoperatively, and the observed phenomena are shown in the middle figure. (Reproduced with permission from the Reference [56]).

The cornea itself does not contain blood vessels, and has an “immune immunity” status, so the success rate of cornea transplantations is high. In recent years, Nishidab et al. developed a new cell sheet manipulation technique that uses temperature-sensitive culture surfaces to generate functional corneal epithelial cells for transplantation. First of all, the humans’ or rabbits’ limbal stem cells were cocultured with a 3T3 feeding layer treated by mitomycin-c in a 37 °C culture dish. After 2 weeks, the temperature dropped below 20 °C and cell sheets were obtained from petri dishes (Figure 20A) [56]. In this technology, complete multilayer corneal epithelial sheets are obtained simply by lowering the temperature without using trypsin. The cell-to-cell connection and extracellular matrix on the basal side of the lamina remain intact, which is critical for the integrity and function of the lamina. After obtaining a thin sheet of corneal cells, the corneal transplant is performed. After the corneal transplantation, fluorescein staining shows that the corneal epithelium evenly covers the entire surface of the cornea, indicating that the operation was successful. On the second day, the sheet is firmly attached to the corneal surface, and some ocular surface inflammation is seen. During the healing process, corneal transparency is restored. The corneal epithelium regenerated after corneal fragment healing is normal in appearance, which is slightly thin, the basal cells are cubic, and the medial and superficial cells are flat. These results indicate that corneal transplantation is successful and prove that bottom-up tissue engineering could be applied to corneal culture.

#### 4.1.4. Myocardial Tube

**Figure 21:**
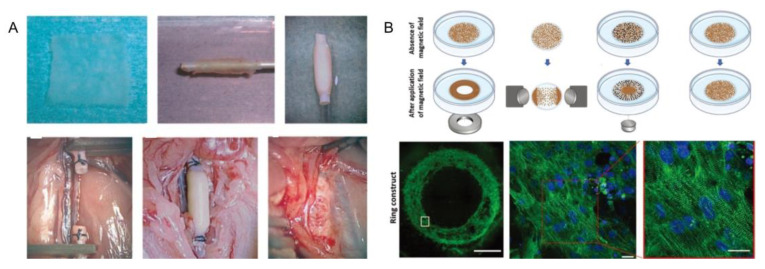
(**A**) Acquisition and transplantation of myocardial tubes. Thin sheets of cells are separated from the temperature-sensitive surface by lowering the temperature, and sheets of cardiomyocytes are wrapped around the resected thoracic aorta. After clamping and resecting the host aorta, the myocardial tube is connected to the host vessel. Four weeks after aortic replacement, the myocardial tube shows fusion with the host tissue. (Reproduced with permission from the Reference [136]). (**B**) Using magnetic poles of different shapes to obtain the corresponding shapes of myocardium sheets for the repair of damaged epicardium. Fluorescence images in different magnetic field configurations. (Reproduced with permission from the Reference [71]).

The myocardium has a risk of losing its systolic function due to ischemia and hypoxia. At present, there are several ways to treat the above diseases [135]. In the present study, Sekine et al. demonstrated the potential of using cell sheet engineering to construct pulsating cardiac tubes for in vivo circulation support (Figure 21A) [136]. Firstly, the thin sheets of cells are separated from the temperature-sensitive surface by lowering the temperature, and then these sheets of cardiomyocytes are wrapped around the resected thoracic aorta. After clamping and resecting the host aorta, the myocardial tube is connected to the host vessel. Four weeks after aortic replacement, the myocardial tube shows fusion with the host tissue. This phenomenon suggests that the myocardial tube made by bottom-up tissue engineering can be transplanted to treat the loss of the myocardial tube or the loss of myocardial tube function.

#### 4.1.5. Epicardium

Subepicardial myocardial injury is a type of coronary heart disease. In many cases of heart failure, a variety of procedures have been used, such as heart transplants [137]; however, this method is also greatly limited, due to a shortage of organs and other problems. Thus the researchers developed bottom-up tissue engineering for regenerating epicardium. A typical example is the use of remotely operated, magnetically assisted assembly to obtain thin cell sheets that can be used to repair the epicardium. Zwi-Dantsis et al. placed the collagen liquid suspension containing the magnetic particle labeled hiPSC-CMs on the center of a petri dish. Next, the cells were exposed to different shapes of external magnets. As the gel solidified, different patterns of epicardium tissue formed (Figure 21B) [138]. In addition, when these cell sheets are transplanted into the damaged heart, they communicate morphologically with the host heart through functional gap junctions and significantly improve cardiac function, which can demonstrate their potential to repair cardiac tissue. The result demonstrates that the epicardium can be fabricated by bottom-up tissue engineering, which can be transplanted to treat myocardial injury. 

#### 4.1.6. Blood Vessel 

**Figure 22:**
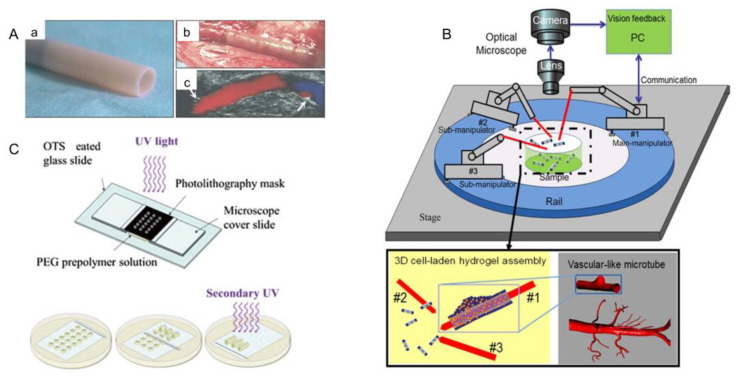
(**A**) Acquisition and transplantation of myocardial tubes. (**a**) Fibroblasts are extracted from patients, and thin sheets of cells are made by using the hydrophobicity of thermosensitive surfaces. The temperature is lowered to separate the thin sheets from the thermosensitive surfaces (**b**), and the resulting thin sheets are wrapped and grown around a cylindrical mandrel to create robust blood vessels (**c**). (Reproduced with permission from the Reference [142]). (**B**) Manufacture of blood vessels by means of robotic assembly. (Reproduced with permission from the Reference [100]). (**C**) Tubular structures are formed by sequential assembly of concentric microgels loaded with cells. Cell-loaded microgels and their assembly (middle). The inner ring is made up of endothelial cells (green) and the outer ring is made up of smooth muscle cells (red) (bottom). (Reproduced with permission from the Reference [143]).

A major limitation of many engineered tissues is the lack of a functional microvascular system to ensure blood perfusion and connection to surrounding tissues. There is more than one bottom-up tissue engineering method that can be used to make blood vessels [139,140]. In earlier studies, human blood vessels could be made by using cell sheet technology (Figure 22A). Fibroblasts were taken from patients, and enlarged sheets of fibroblasts were wrapped and grown around cylindrical mandrels to create robust blood vessels [141]. However, the geometry that can be made with thin plates is limited, and the connections between layers are inadequate. Another approach is to use robotic arms to complete the assembly of blood vessels based on the pickup and placement function of microrobots. This method has high precision and can be used to assemble heterogeneous modules, but it also has the problem of inefficiency (Figure 22B) [100]. In addition to the above two methods, it is also possible to fabricate blood vessels by sequentially assembling concentric microgels carrying cells to form tubular structures (Figure 22C) [31]. Du et al. demonstrated the feasibility of continuous assembly of concentric cell-loaded microgel modules to form tubular structures. Each microgel module unit consists of two concentric hydrogel rings loaded with two different types of cells. The manufacturing process of these cell-loaded microgel modules involves two types of photolithography. First, the inner ring filled with endothelial cells is made, followed by the outer ring filled with smooth muscle cells. These concentric microgels were assembled into tubular structures and further stabilized by the application of secondary violet couplets. This modular approach has shown good results. The above three methods can prove the feasibility of bottom-up tissue engineering to manufacture blood vessels.

#### 4.1.7. Adipose Tissue 

**Figure 23:**
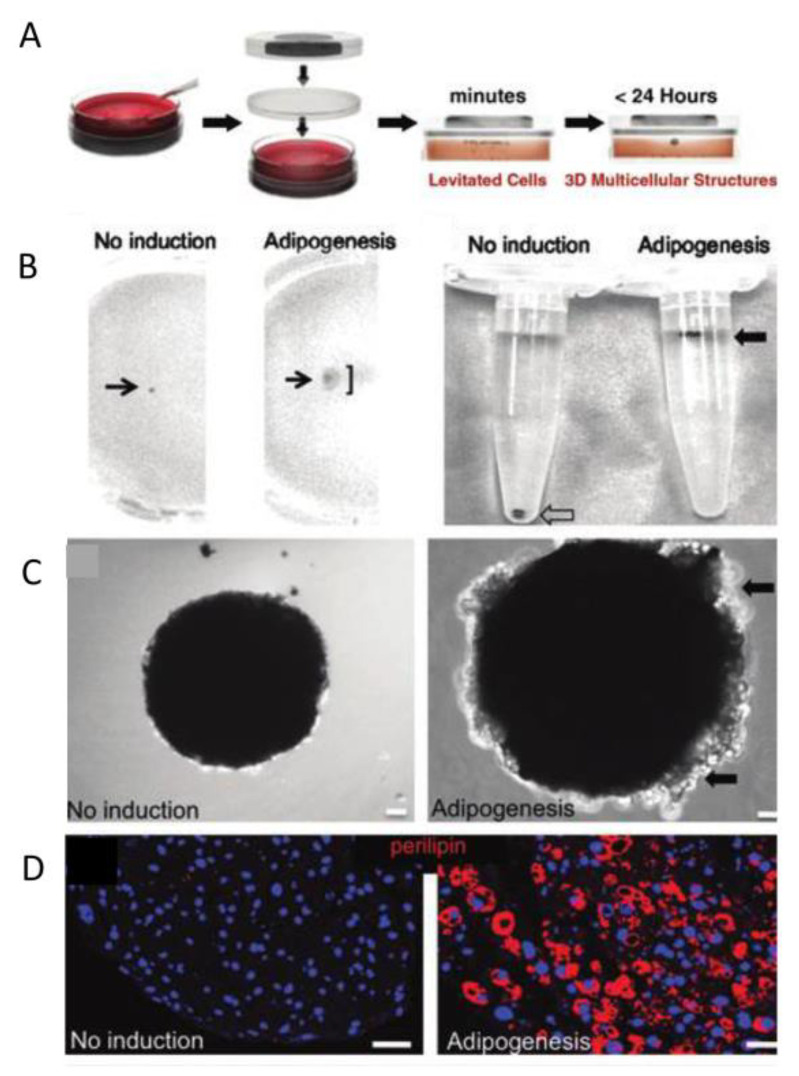
Construct adipose tissue by magnetic-assisted assembly. (**A**) The cells are labeled with magnetic particles and placed in a petri dish, and then a magnet is placed above the dish. At this point, the cells begin to float below the liquid level (up). Adipocytes are cultured for 14 days without induction (left) and under adipogenesis induction (right). (**B**) Adipocyte spheres show buoyancy in phosphate-buffered saline 14 days after lipid accumulation induced adipogenesis (right tube, solid arrow). The left tube shows a control sphere (no buoyancy, hollow arrow) at the bottom of the tube without induced culture. (**C**) The cells are suspended for 8 days in the absence of induction and in the presence of adipogenesis induction, respectively, and the adiposphere begins to form, surrounded by fat cells containing large lipid droplets (arrows). (**D**) Marked with phospholipid antibody immunofluorescence (red), indicating lipid droplet maturation in adipocytes composing the spheroid upon culture in the adipogenesis induction medium. (Reproduced with permission from the Reference [145]).

Adipose tissue is a mass of fat cells separated into lobules by thin layers of loose connective tissue [144]. Adipose tissue also plays an important role in metabolism. White adipose tissue (WAT) is increasingly used in regenerative medicine and cell therapy, whose physiological and pathological importance is gaining increasing attention. Thus it is very important to grow adipose tissue in vitro, and several methods of in vitro culture have been developed. Daquinag et al. used a magnetic levitation system to grow adipose tissue [144,145]. Cells and magnetic particles were placed into a culture plate well and a magnet driver was placed above this well, and cells then levitated to the meniscus. Cell aggregates assembled into a sphere, which were cultured without induction (left) or fat formation induction (right) for 14 days (Figure 23A). The tube on the right shows the fat sphere cultured with induction suspended below the liquid level (with buoyancy, solid arrow). The tube on the left shows a control sphere (no buoyancy, hollow arrow) at the bottom of the tube cultured without induction (Figure 23B). The fat sphere cultured with induction was suspended by the action of buoyancy, and this phenomenon was due to lipid accumulation. When suspended for 8 days, the fat sphere began to form, surrounded by fat cells containing large fat droplets (arrows). After 14 days of fat formation, the sphere in the induced medium became significantly larger and the surrounding fat cells could be seen by phase contrast microscopy (Figure 23C). Marked with phospholipid antibody immunofluorescence (red), it showed the lipid droplet maturation of globular adipocytes in the culture medium for inducing adipogenesis (Figure 23E). These results show that 3D suspension adipose tissue culture based on the assembly of magnetic nanoparticles is feasible, and the importance of induction in the culture of adipose tissue by this method is proven. This research is an important application of bottom-up tissue engineering in tissue culture.

### 4.2. Drug Screening

Drug development is a time-consuming and costly process. With costs increasing exponentially, especially in the late development stages of clinical studies, the failure of a drug after late development or approval is the worst-case scenario. For instance, many cardiac side effects, such as arrhythmias, are the most common reason for drug development suspension [146]. In the application of cardiac medicine, approximately 45 percent of withdrawals and 30 percent of drug limitations are due to cardiovascular side effects. Clinically, about 70% of drugs can be screened at the early clinical stage. If the predictive value of preclinical testing is increased by 10 percent, the average cost of research and development for each drug would be saved by $100 million. With the development of tissue engineering, bottom-up tissue engineering has been used in drug screening. 

**Figure 24:**
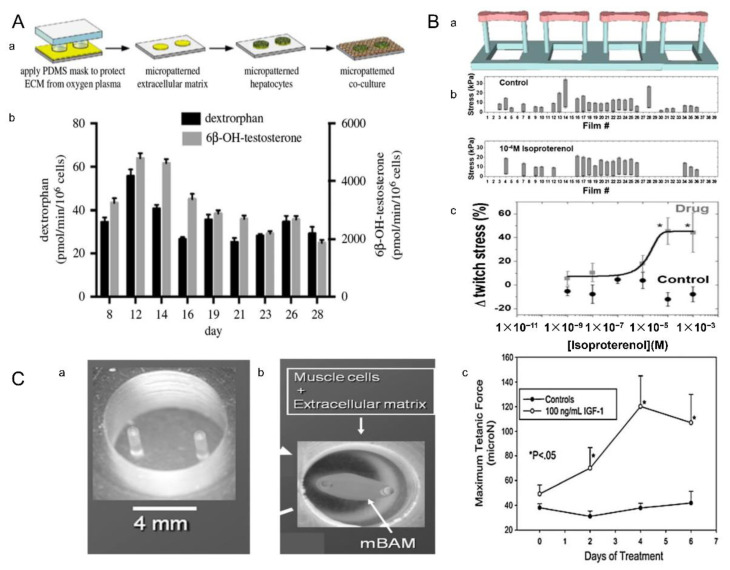
(**A**) (**a**) Building a liver model. Micromode coculture (MPCCs) allows the regulation of homotypic interactions between hepatocytes and heterotypic interfaces between hepatocytes and fibroblasts, while maintaining a constant cell count/ratio in various mode configurations. (**b**) Tissue cultured by MPCCs can maintain a high level of CYP450 activity for several weeks, which can greatly reduce the influence of culture time on CYP450 activity. (Reproduced with permission from the Reference [148]). (**B**) (**a**) Mechanical drawing of a silicone rack with four pairs of posts and EHTs spanning between them. (**b**) The control group without applying the drug. The other group is an experimental group that inflicted the isoproterenol. The lower y-intercept in the two bars represents the relaxation stress, and the higher y-intercept represents the peak contraction stress. The length of the bar chart represents the heart’s ability to contract and relax. (**c**) Twitch stresses for each MTF from this chip are plotted. (Reproduced with permission from the Reference [150]). (**C**) (**a**) Engineering screening of musculoskeletal tissue for high drug content. (**b**) mBAM showed the structure at days 4 to 5 of culture. (**c**) Insulin growth factor-1 (IGF-1) on muscle strength synthesis. Insulin growth factor was added 6–7 days after mBAM differentiation. (Reproduced with permission from the Reference [149]).

Heteromorphic interactions between liver parenchymal cells and nonparenchymal cells are crucial in liver development [147], so the coculture of 3T3-J2 fibroblasts with primary hepatocytes has been widely used in drug development (Figure 24A(a)) [148]. Rifampicin is a broad-spectrum antibiotic drug, which has a strong antibacterial effect on tuberculosis bacilli, and also has a curative effect on gram-positive or -negative bacteria and viruses [149,150,151]. Rifampicin can activate the CYP enzyme, which is known as cytochrome oxidase P450, and is a group of isoenzymes encoded by superfamily genes related to structure and function. CYP3A4 and CYP2D6 account for a large proportion of CYP enzymes, and their activities can also be activated by rifampicin. The activity of CYP3A4 (cytochrome P4503A4 enzyme) can be assessed by metabolizing testosterone into 6B-OH-testosterone. Tissue cultured by MPCCs (micropattern co-culture) can maintain a high level of CYP2D6 (cytochrome P450 isozymes) activity for several weeks, which can greatly reduce the influence of culture time on CYP2D6 activity, ensuring that changes in CYP2D6 activity were caused by rifampicin (Figure 24A(b)). Then the activity of CYP2D6 (cytochrome P450 isozymes) can be assessed by the metabolism of dextromethorphan into coffee alkanes. Finally, the efficacy of rifampicin can be evaluated by testing the content of metabolites.

Eder et al. prepared the myocardium by using the bottom-up method [152]. The annular heart tissue was nested on the two pillars, and the cardiomyocytes grew toward the pillars over time. After one week of culture, the tissue became contractible and developmentally contracted against the elastic resistance of the pillars (Figure 24B(a)). Isoproterenol is a non-selective β-adrenergic agonist, which has obvious characteristics in the treatment of bradycardia. Agarwal et al. tested the in vitro effects of isoproterenol on electrical stimulation of cardiac microtissue in a microdevice. In this set of experiments, the control group did not apply the drug. The other group was an experimental group that inflicted the isoproterenol (Figure 24B(b)) [153]. The lower y-intercept in the two bars represents the relaxation stress, and the higher y-intercept represents the peak contraction stress. The length of the bar chart represents the heart’s ability to contract and relax. The bars in the control group were shorter than those in the experimental group, suggesting that isoproterenol can be used to treat bradycardia by increasing the systolic and diastolic capacity of the heart. The twitch pressure of the heart is also an important parameter to describe the contract ability of the heart. It can be seen from the graph that the twitch pressure increased when the adrenaline content of the experimental group moderately increased. However, the twitch pressure in the control group was always lower than that of the experimental group, and the value almost remained stable, which also proved the applicability of epinephrine in the treatment of bradycardia (Figure 24B(c)).

In a past study, Vandenburgh et al. screened the efficacy of insulin-like growth factor-1 (IGF-1) by testing the mechanical tests of musculoskeletal organs. Cell-loaded microgel module units were cultured in molds for 7–8 days to obtain annular muscle tissue (Figure 24C(a)) [132,149], and then the insulin growth factor was added after 6–7 days of culture. The ringed muscle tissue was then attached to two columns molded from flexible polydimethylsiloxane (Figure 24C(b)). Then, drug screening could be carried out by observing the relationship between the maximum tonic force and insulin factor (Figure 24C(c)). It was observed that the muscle maximum tetanic force of the experimental group increased, while that of the control group was smaller and almost unchanged. These results showed that the insulin growth factor-1 can enhance the ability of muscle contraction and relaxation, which can be used to treat muscle diseases. This research proved that bottom-up tissue engineering has a wide range of clinical application value in the treatment of muscle diseases.

### 4.3. Cancer Treatment

**Figure 25:**
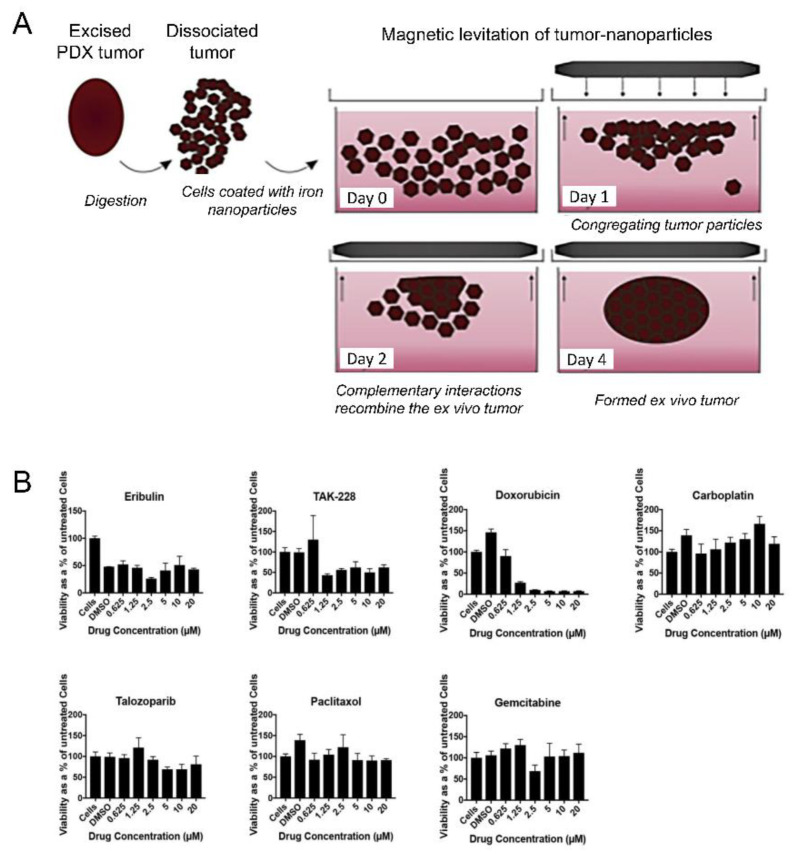
(**A**) Acquisition of human tumor allograft model (PDXEx). PDX tumors freshly harvested from mice are finely chopped to release all their cellular contents. The released cells are filtered, then labeled with magnetic particles and placed in a magnetic field overnight. Floating cell masses developed into loose unstructured masses on two days of incubation. They develop into more structured, compact clumps on the fourth day. (**B**) Effects of several anticancer drugs on the activity of cancer cell aggregates. (Reproduced with permission from the Reference [154]).

Bottom-up tissue engineering is not only widely used in tissue manufacturing and drug screening, but also provides a new technological approach for studying the mechanisms of angiogenesis and tumor cell growth [148,151,152,153]. Inflammatory breast cancer (IBC) is a rare special type of breast cancer [148,154,155]. Currently, researchers are using bottom-up tissue engineering for cancer treatment. The most clinically effective model is the patient-derived xenotransplantation (PDX) model, which reproduces the heterogeneity of the patient’s tumor and demonstrates the genetic stability of the closely stable replication of the human tumor microenvironment. However, the generation of the PDX model requires a lot of resources, and is high-cost and time-consuming [156]. Thus, the researchers demonstrated an in vitro (PDXEx) model of IBC PDX derived from the cellular environment released by mouse PDX tumors, which were similar to that of the original PDX tumor. The PDXEx model was made by using the magnetic assembly method (Figure 25A) [154]. Firstly, breast cancer tumors taken from mice were chopped up so that the contents were released completely. The released cells were filtered and then labeled with iron oxide and poly-L-lysine crosslinked iron nanoparticles (Nanoshuttle™) to make them magnetic. A culture medium containing cells was put under a floating magnet. The cells formed loose unstructured clumps on the second day, and formed more structured, compact clumps on the fourth day. Finally, an in vitro model for cancer screening was obtained. Additionally, the constructed tissue was treated with several kinds of drugs in a specified dose range for 5 days (Figure 25B). By comparing the effects of different drugs on the activity of breast cancer cells, the most suitable drugs for the treatment of breast cancer can be selected. These results confirm the value of the PDXEx model in identifying effective tumor-specific therapies.

## 5. Conclusions

Bottom-up tissue engineering has developed rapidly and become a popular research direction in the tissue engineering field. Recently, bottom-up tissue engineering has been widely used in the areas of tissue reconstruction, drug screening and cancer treatment. However, many bottom-up assembly methods are just preliminary explorations. There are still many problems which need to be solved in order to achieve large-scale promotion and clinical applications. Although some modular units with specific shapes and components can be prepared by using current methods, there is still a large gap between the structure and function of such modular units and functional units in the human body. Tissues or organs in the human body are not simply a repeated accumulation of functional units, and the connections between living blocks have an important impact on the biogenic, mechanical-electrical behavior of systematic tissues or organs. Therefore, the assembly process of modular units needs to be more accurately regulated. Vascularization is not only a bottleneck in the development of tissue engineering, but also a challenging problem that must be solved in the development of bottom-up tissue engineering technology. Although the current methods can achieve the construction of a certain structure of the bionic vascular network, the structure and function of the real vascular network are still far from that of the real vascular network. Chemical stimulation and mechanical stimulation also play an important role in tissue or organ formation and functionalization. Therefore, the controllable chemical and mechanical control of modular units and three-dimensional assembly structures is a problem that needs further study. Like traditional tissue engineering technology, the ultimate goal of bottom-up tissue engineering technology is to achieve tissue or organ transplantation. However, the compatibility of such engineered tissues with the environment of the human body, the matching ability of mechanical properties, and the connectivity of the vascular network need to be solved after transplantation. In short, with the development of micro-nano biological manufacturing technology and the emergence of new biological materials, bottom-up tissue engineering technology will definitely promote the development of tissue engineering and regenerative medicine and move toward the goal of transplantable tissues or organs, forming a far-reaching medical revolution. At the same time, bottom-up tissue engineering technology will also be greatly developed and applied in the study of pathophysiological mechanisms and drug screening based on an in vitro model. We envision that a series of new enabling technologies can promote the development of modular assembly of more complex tissue targets.

## Figures and Tables

**Table 1:** Summary of the advantages and disadvantages of methods for module unit preparation.

Module Manufacturing Method	Advantages	Disadvantages	Reference
Emulsion method	- Easy to produce microgels.	- Larger size distribution in gel. - Little control over the shape.	[23,25]
Optical graphicsmethod	- Easy to control shape and size. - Easy to generate microgels. - Programmable. - High throughout.	- Affect cell activity. - Only for photosensitive materials.	[26,27]
Microchannelmethod	- Control the shape of micgels. - Continuity. - Can be used combine with other- methods.	- Need precisely designed microchannels and complex devices. - Low-throughout. - Need rapid prototyping of materials.	[28,29,30,31,32,33,34,35]
Liquid bridgemethod	- Unrestricted material.	- Evaporation.	[36,37,38]
Microporous platemethod	- Wide range of application. - Simple.	- Heterogeneity. - Hydrogels may expand upon release, resulting in a different size from the PDMS mold.	[39,40]
Bioprintingtechnology	- Fast; - The survival rate of cells is high; - High precision.	- Expensive. - Resolution is limited.	[41,42,43,44]

**Table 2:** The advantages and disadvantages of the eight assembly methods of module units.

Assembly Method	Advantages	Disadvantages	Reference
Surface modification	- Fast and scalable; - Large size; - No support; - Simple operation.	- Lack of 3D complex structure; - Limited cell type; - Low structural controllability; - Low spatial resolution.	[1,25,49,52,53,54,55,56,57,58,59,60,61,62,63,64,65]
Magnetic assembly	- Simple operation; - High controllable accuracy.	- Cell activity is affected; - Magnetic field attenuation affects 3D structure.	[66,67,68,69,70,71]
Acoustic assembly	- Fast assembly speed; - Keep cells active.	- Acoustic wave attenuation affects 3D structure; - Lack of 3D complex structure.	[72,73,74,75,76,77,78,79,80,81,82]
Dielectrophoresisassembly	- Fast.	- Cell activity is affected.	[45,83,84,85,86,87,88,89,90,91,92,93]
DNA assistedassembly	- Accurate and reliable; - Multiplexing.	- Form complex three-dimensional structures difficultly.	[42,94,95,96,97]
Robot assembly	- High precision; - Allows assembly of - heterogeneous materials.	- Low-throughout.	[31,98,99,100,101,102]
Optical assembly	- Accurate and reliable; - Can be used for single cell construction.	- Cell activity is affected	[80,103,104,105]
Microfluidic method	- Simple operation; - Flexible; - High-throughout.	- High production costs.	[11,25,28,47,98,106,107,108,109,110,111,112,113,114,115,116,117,118,119]

## Data Availability

Data sharing is not applicable to this article as no datasets were generated or analysed during the current study.
